# Size Does Matter: An Integrative *In Vivo*-*In Silico* Approach for the Treatment of Critical Size Bone Defects

**DOI:** 10.1371/journal.pcbi.1003888

**Published:** 2014-11-06

**Authors:** Aurélie Carlier, Nick van Gastel, Liesbet Geris, Geert Carmeliet, Hans Van Oosterwyck

**Affiliations:** 1 Biomechanics Section, KU Leuven, Leuven, Belgium; 2 Prometheus, Division of Skeletal Tissue Engineering, KU Leuven, Leuven, Belgium; 3 Biomechanics Research Unit, University of Liège, Liège, Belgium; 4 Clinical and Experimental Endocrinology, KU Leuven, Leuven, Belgium; Johns Hopkins University, United States of America

## Abstract

Although bone has a unique restorative capacity, i.e., it has the potential to heal scarlessly, the conditions for spontaneous bone healing are not always present, leading to a delayed union or a non-union. In this work, we use an integrative *in vivo* - *in silico* approach to investigate the occurrence of non-unions, as well as to design possible treatment strategies thereof. The gap size of the domain geometry of a previously published mathematical model was enlarged in order to study the complex interplay of blood vessel formation, oxygen supply, growth factors and cell proliferation on the final healing outcome in large bone defects. The multiscale oxygen model was not only able to capture the essential aspects of *in vivo* non-unions, it also assisted in understanding the underlying mechanisms of action, i.e., the delayed vascularization of the central callus region resulted in harsh hypoxic conditions, cell death and finally disrupted bone healing. Inspired by the importance of a timely vascularization, as well as by the limited biological potential of the fracture hematoma, the influence of the host environment on the bone healing process in critical size defects was explored further. Moreover, dependent on the host environment, several treatment strategies were designed and tested for effectiveness. A qualitative correspondence between the predicted outcomes of certain treatment strategies and experimental observations was obtained, clearly illustrating the model's potential. In conclusion, the results of this study demonstrate that due to the complex non-linear dynamics of blood vessel formation, oxygen supply, growth factor production and cell proliferation and the interactions thereof with the host environment, an integrative *in silico*-*in vivo* approach is a crucial tool to further unravel the occurrence and treatments of challenging critical sized bone defects.

## Author Summary

In 5–10% of fracture patients, the bone fractures do not heal in the normal healing period (delayed healing) or do not heal at all (non-union). In order to investigate the causes of impaired healing and design potential treatment strategies, we have used a combined experimental and computational approach. More specifically, large bone defects were analyzed in mouse models and simulated by a previously published computational model. After showing that the predictions of the computational model match the observations of the experimental model, we have used the computational model to investigate the underlying mechanisms of action. In particular, the results indicated that the new blood vessels do not reach the central fracture zone in time due to the large defect size, which leads to insufficient oxygen delivery, increased cell death and disrupted bone healing. The healing, however, could be rescued by adequate blood vessel ingrowth from the overlying soft tissues. Moreover, potential treatment strategies were designed based on the influence of these soft tissues. In conclusion, this study demonstrates the potential of a combined experimental and computational approach to contribute to the understanding of pathological processes like the impaired bone regeneration in large bone defects and design future treatments thereof.

## Introduction

Although bone has a unique restorative capacity, i.e. it has the potential to heal scarlessly, the conditions for spontaneous bone healing are not always present, leading to a delayed union or a non-union. The orthopedic literature does not specify a universally accepted definition of a fracture non-union [1], [2]. The eventual bony union after an atypical long period of healing, in comparison to the normal healing period, is called a delayed union. The absence of healing during at least three to six months defines a fracture non-union in humans. Fracture non-unions (hypertrophic, atrophic or oligotrophic) are classified based on their radiographic and histological appearance [1], [3].

Hypertrophic non-unions are characterized by an abnormal vascularity and abundant callus formation. They are typically caused by excessive motion at the fracture site, which prevents bony bridging although the essential biological factors are present [1]. Atrophic non-unions, however, are the result of inadequate biological conditions and typically appear on radiographs as blunted bony ends. They show little callus formation around the fracture gap, filled with mostly fibrous tissue and little or no evidence of mineral deposition [1]. Oligotrophic non-unions have some radiographic and biological characteristics of both hypertrophic and atrophic non-unions, i.e. they possess the required biological activity but show little or no callus formation [4].

Excess motion, a large interfragmentary gap [5], open fracture [5]–[7], the particular bone [8], location of the trauma within the bone [8], loss of blood supply [9], severe periosteal and soft-tissue trauma [6], [7] are some of the mechanical and biological risk factors for the development of a non-union. Preexisting patient risk factors such as old age [10], cachexia and malnutrition [11], immune compromise [12], genetic disorders (e.g. type 1 neurofibromatosis), osteoporosis [13], anticoagulants [14], anti-inflammatory agents [15], etc. may also affect the fracture healing outcome but are not the primary causes [16]. Besides an extensive amount of experimental research, several computational models have also been developed to further unravel the occurrence of fracture non-unions. For comprehensive reviews on mathematical models of fracture healing, we refer the reader to Geris et al. [17], Isaksson et al. [18] and Pivonka et al. [19]. Despite the large amount of (often phenomenological) information existing in the literature, additional *in vivo*, *in vitro* and *in silico* research is still required to address the key mechanisms that lead to fracture non-unions, determine the factors predictive of fracture complications and establish the optimal therapeutic strategies for each type of fracture non-union.

In this work we propose an integrative *in vivo* - *in silico* approach to investigate the occurrence of oligotrophic and atrophic non-unions as well as to design possible treatment strategies thereof. The gap size of the domain geometry of a previously published mathematical model has been enlarged in order to study the complex interplay of blood vessel formation, oxygen supply, growth factors and cell proliferation on the final healing outcome in large bone defects. The simulation results are corroborated by comparison with dedicated experimental data. Next, the mathematical model is used to explain the underlying mechanisms that lead to the experimental observations as well as design different treatment strategies. Finally, the potential of the combined *in silico* - *in vivo* approach is demonstrated by applying it to the case of BMP-treated fracture healing.

## Materials and Methods

### Ethics statement

All animal experiments were conducted according to the regulations and with approval of the Animal Ethics Committee of the KU Leuven.

### Animals and operative procedure

C57BL/6 mice were purchased from the R. Janvier Breeding Center (France). A segmental defect was created in the right tibia of 14 week-old male mice as described elsewhere [20]. Briefly, animals were anaesthetized with a ketamine-xylazine mixture (100 mg/kg ketamine and 15 mg/kg xylazine) and the right lower leg was shaved. A custom-made external fixator, based on the Ilizarov external fixation device, was fixed to the tibia using 27 G steel needles. Subsequently, the tibia was exposed and a 4.5–5 mm mid-diaphyseal segment was excised with a 6.5 mm diamond saw disk (Codema n.v., Kortrijk, Belgium). A demineralized CopiOs scaffold (2.5×2.5×5 mm^3^; Zimmer b.v.b.a., Wemmel, Belgium) seeded with 1×10^6^ mouse periosteal cells (passage 4) was implanted, the skin was sutured and animals received postoperative analgesia (buprenorphine, 60 µg/kg body weight). The demineralized CopiOs- scaffold was used to minimize the soft tissue collapse within the critical size defect. After 3, 14 or 56 days animals were sacrificed, the tibia was excised and samples were analyzed by μCT and then processed for histology.

### Isolation and culture of murine periosteum-derived cells

Murine periosteum-derived cells (mPDC) were isolated from the long bones of 8 week-old male mice as previously described [21]. In short, the femurs and tibias isolated from 8 week old male C57BL/6J mice were dissected and digested with collagenase-dispase after protecting the epiphyses with low melting point agarose. After a filtration and washing step, the cells were plated at 1×10^4^ cells per square centimeter and replated when reaching 80–90% confluency. After isolation, cells were pooled per 2–3 mice and cultured in a humidified incubator at 37°C with 5% CO_2_ in α-minimal essential medium (α-MEM) supplemented with 2 mM glutaMAX-I, 1% penicillin/streptomycin (100 units/ml and 100 µg/ml respectively) and 10% fetal bovine serum (all from Gibco, Life Technologies, Gent, Belgium). When reaching 80–90% confluency, cells were trypsinized and reseeded at 7500 cells/cm^2^.

### Transduction of mPDC

To deliver BMP2 at the defect site, mPDCs were transduced 72 hours prior to implantation with an adenoviral vector encoding human BMP2 (a generous gift from Dr. Frank Luyten, KU Leuven, Belgium) at a multiplicity of infection of 50.

### Radiographic and μCT analyses

Bone formation in large bone defects was followed by radiographic images at different time points after surgery using the Skyscan 1076 high resolution *in vivo* micro-computed tomography (μCT) scanner (Bruker-μCT, Kontich, Belgium). For bone quantification, samples retrieved at day 56 were scanned using the high resolution SkyScan 1172 μCT system (Bruker-μCT) at a pixel size of 10 µm with 50 kV tube voltage and 0.5 mm aluminum filter. Projection data was reconstructed using the NRecon software and quantification of mineralized tissue was performed using the CTAn software (both from Bruker-μCT).

### Histology and immunohistochemistry

Isolated bones were fixed in 2% paraformaldehyde overnight and decalcified in EDTA for 14 days at 4°C prior to dehydration, embedded in paraffin and sectioned at 4 µm. Histochemical staining with hematoxylin and eosin (H&E) and immunohistochemical staining for mouse CD31 is routinely performed in our lab and has been described previously [21]. Images were taken on a Zeiss Axioplan 2 light microscope using the Zeiss AxioVision software.

### Statistical analysis

Data are presented as means ± standard error of the means. Data were analyzed by one-way ANOVA using the NCSS statistical software. Differences were considered statistically significant at p<0.05.

### Mathematical model

**Figure 1:**
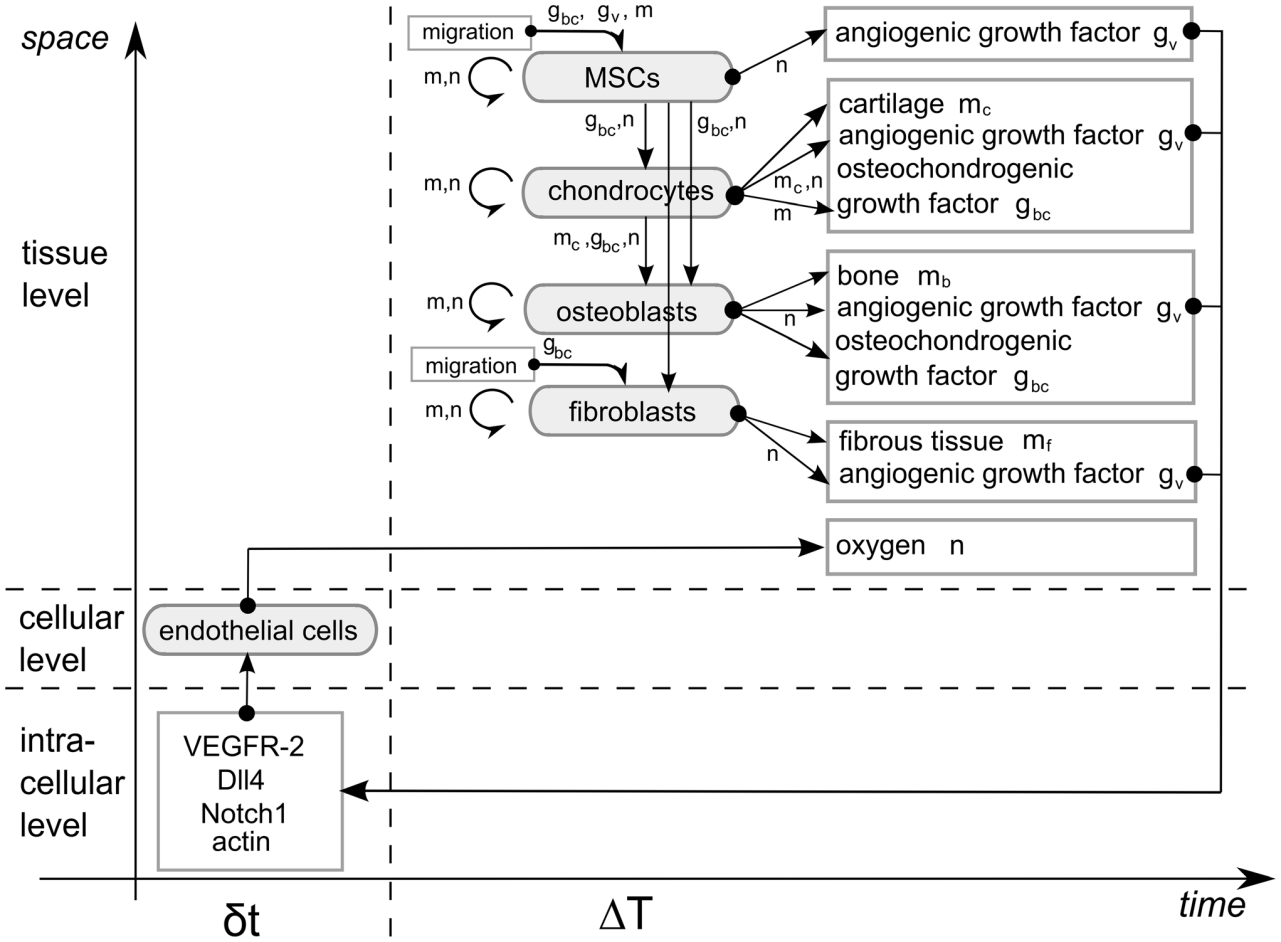
Schematic representation of the multiscale oxygen model. *m = m_f_+m_c_+m_b_* represents the total tissue density. The intracellular variables govern the endothelial cell (EC) behavior. At the tissue scale, cells can migrate (only MSCs and fibroblasts), proliferate (circular arrows), differentiate (vertical arrows), produce growth factors and extracellular matrix. Blood vessels are a source of oxygen which influences proliferation, differentiation and hypoxia-dependent angiogenic growth factor production. Variables influencing a tissue level process are indicated next to the corresponding arrow.

The multiscale computational framework for the mathematical modelling of bone fracture healing and its relation to angiogenesis was established earlier and has been described in detail in [22]. The framework consists of (1) a tissue level describing the various key processes of bone fracture healing with 10 continuous variables, (2) a cellular level representing the developing vasculature with discrete endothelial cells and (3) an intracellular level that defines the internal dynamics of the Dll4-Notch signaling pathway in every endothelial cell (Figure 1). The model accounts for the various key processes that occur during the soft and hard callus phase of bone fracture healing (see [22] for a more detailed description). While the model described in [22] already partially accounted for the role of oxygen, we have recently extended the model to capture the various effects of oxygen on cellular processes in a much more complete and refined way [23]. A brief description of the oxygen model is found below and more details are given in Supporting Text S1.

After the initial inflammation phase (which is not included in the current mathematical model), the fracture callus is filled with a cocktail of granulation matrix, stem cells and growth factors. In regions where oxygen is abundantly available (i.e. close to the cortex in the case of normal fracture healing), the mesenchymal stem cells will directly differentiate into osteoblasts and form bone through the intramembranous pathway. In regions where the oxygen tension is lower (i.e. the central fracture callus in the case of normal fracture healing), the mesenchymal stem cells will differentiate to chondrocytes that will form a cartilage template to mechanically stabilize the fracture. This is followed by endochondral ossification during which blood vessels and osteoblasts are attracted to the central fracture callus, resulting in degradation of the cartilage template and bone formation. Finally, the newly formed bone is remodeled (not included in the current mathematical model).

**Figure 2:**
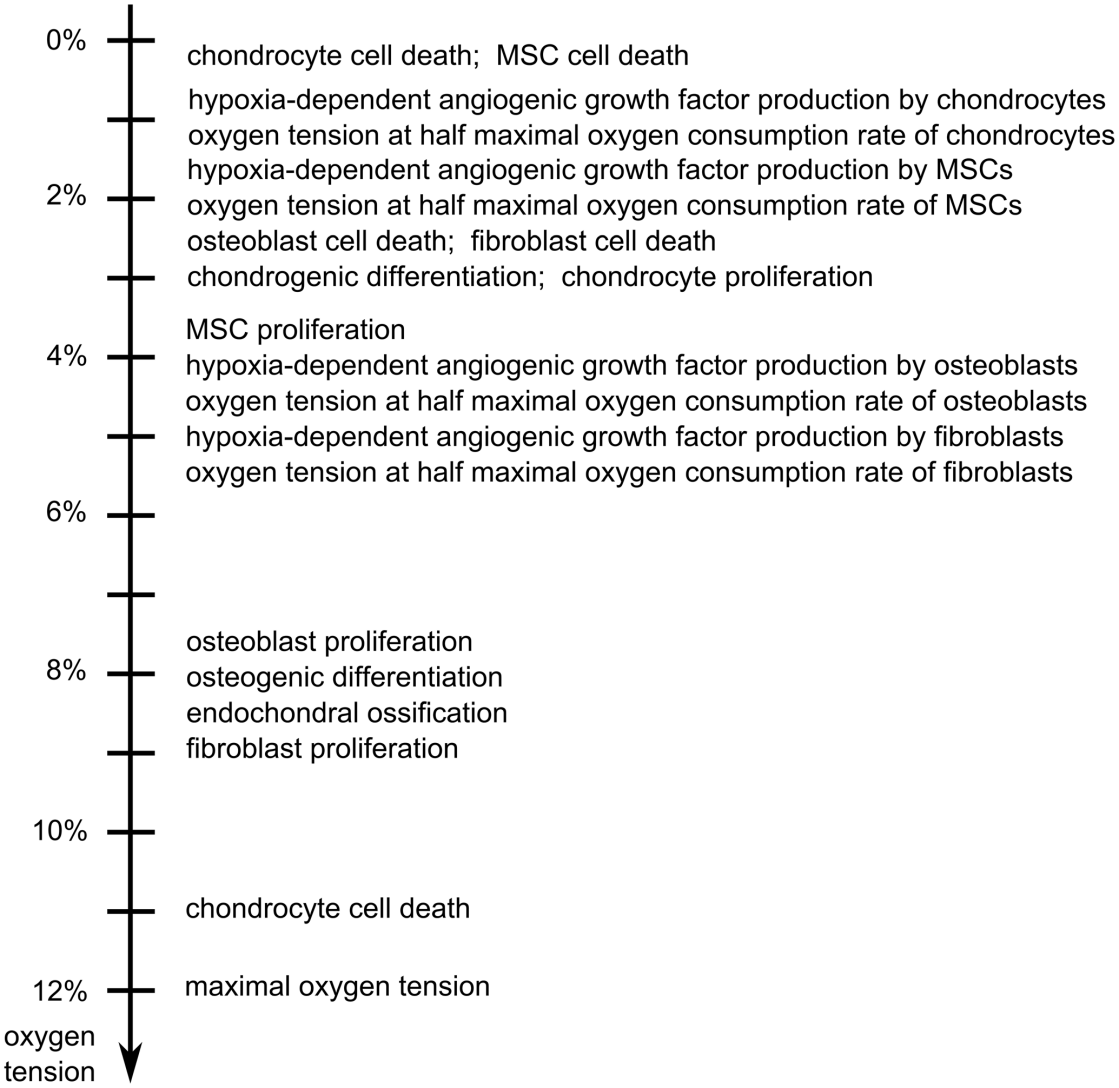
Overview of the oxygen tensions at which the rate of distinct cellular processes is maximal or changes. Biological processes that preferentially take place in low oxygen tensions (upper part of Figure 2) will occur in regions where the oxygen tensions will have dropped with respect to the initial value (e.g. the central fracture zone) while biological processes that preferentially take place in high oxygen tensions (lower part of Figure 2) will occur in regions where the oxygen tensions will have increased with respect to the initial value (e.g. near the blood vessels of the periosteal layer). The initial oxygen tension in the central fracture zone is 3.7%.

At the tissue level, the fracture healing process is described by calculating the spatiotemporal evolution of the density of mesenchymal stem cells (*c_m_*), osteoblasts (*c_b_*), chondrocytes (*c_c_*), fibroblasts (*c_f_*), bone (*m_b_*), cartilage (*m_c_*), fibrous matrix (*m_f_*), osteochondrogenic growth factor (*g_bc_*), angiogenic growth factor (*g_v_*) and oxygen (*n*) using 10 non-linear, coupled partial differential equations of the taxis-diffusion-reaction type. At the cellular level, the evolution of the discrete vasculature is determined by sprouting, vascular growth and anastomosis and is modeled by a lattice-free method. At the intracellular level, an agent-based model is used to implement the rules that capture the intracellular dynamics of the Dll4-Notch signaling pathway which determines tip cell selection during sprouting angiogenesis. The oxygen model includes an accurate description of the oxygen dependency of a number of cellular processes, namely osteogenic and chondrogenic differentiation, cell proliferation, cell death, oxygen consumption and the hypoxia-dependent production of an angiogenic growth factor. The cellular consumption of oxygen was described using a Michaelis-Menten kinetic law where the cell-specific maximal oxygen consumption rate has the following relative cellular order: chondrocytes<MSCs<osteoblasts<fibroblasts. The oxygen values at which the considered cell-specific oxygen-dependent processes occur at maximal rate or at which their rate changes are based on a rigorous literature screening of the state-of-the-art experimental knowledge (Figure 2). More specifically, the relative order of the oxygen dependent processes was determined as accurately as possible since it is crucial to the behavior of the oxygen model. The complete description of the set of equations, the boundary and initial conditions, the parameter values, implementation details as well as some underlying assumptions and simplifications can be found in Supporting Text S1 as well as in previous publications [22], [24], [25].

**Figure 3:**
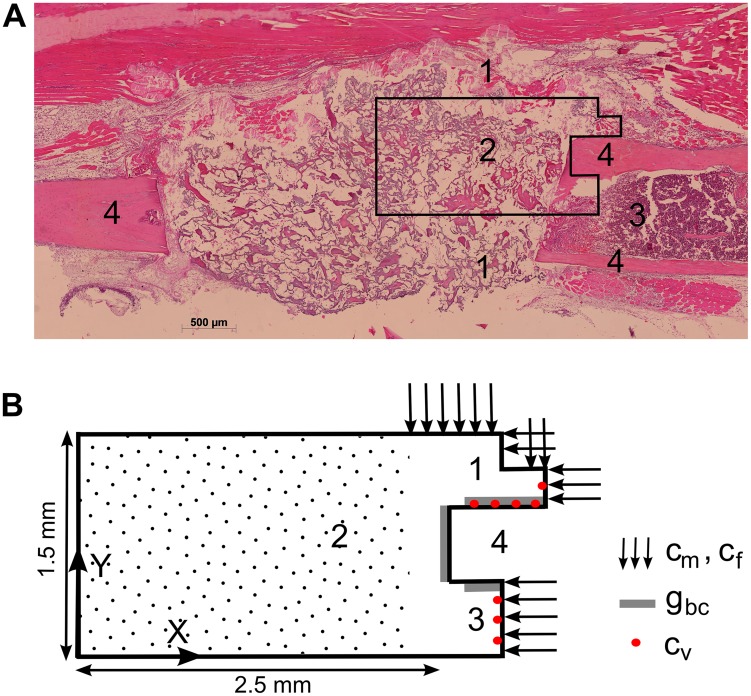
Geometrical domain and boundary conditions of the computational model. (A) The geometrical domain considers one-fourth of the real fracture callus geometry of a critical size defect (assuming symmetry); 1 periosteal callus; 2 intercortical callus; 3 endosteal callus; 4 cortical bone ends. (B) No-flux boundary conditions are assumed for all variables, except for the mesenchymal stem cells (*c_m_*) and fibroblasts (*c_f_*) which are released from the periosteum and surrounding soft tissues near the bony ends as well as the bone marrow [55]; and the osteochondrogenic growth factor (*g_bc_*) which is released from the degrading bone ends and the cortex [56], [57]. The central callus area (indicated with dots) is initialized with a reduced amount of cells (*c_m,init_* = 2.10^3^ cells/ml) and growth factors (*g_bc,init_* = 10 ng/ml) with respect to the tissues surrounding the bone end (*c_m,init_* = 2.10^4^ cells/ml, *g_bc,init_* = 100 ng/ml). The initial oxygen tension (*n_init_*) in the central callus area is 3.7%. The origin of the coordinate system is placed in the left bottom corner of the geometrical domain.

The geometrical domain of the fracture callus, as well as the boundary conditions and initial positions of the endothelial cells (*c_v_*) are shown in Figure 3-B. Note that the periosteum near the bone ends is considered to be well vascularized such that a muscular contribution to the vasculature (i.e. the initial position of the endothelial cells) is unnecessary. To simulate the bone regeneration process in a large bone defect, the domain was extended over a distance equal to half the gap size of a murine critical sized defect (5 mm). The effect of the host environment on the fracture healing process is explored with several combinations of boundary conditions, however in the standard compromised condition the influence of the host environment is neglected thereby representing the worst-case scenario (Figure 3-B). It has been shown experimentally that the amount of cells and growth factors is significantly reduced in a large fracture gap [26], [27]. Therefore, in order to simulate this effect, the initial conditions for the MSCs and osteochondrogenic growth factors were decreased tenfold to 2.10^3^ cells/ml and 10 ng/ml respectively in the central callus area (indicated with dots in Figure 3-B). The initial oxygen tension (*n_init_*) in the central callus area is equal to 3.7%. All other model parameters as well as initial and boundary conditions were left unchanged with respect to the normal healing case [23] and can be found in Supporting Text S1 (Figure 3). Note that the computational model does not simulate the presence of the demineralized CopiOs scaffold, which was used to minimize the soft tissue collapse within the critical size defect. Previous results have however shown that the demineralized carrier structure does not contribute nor enhance the bone formation process.

The results of the mathematical model are quantified in terms of tissue fractions, specified for each part of the fracture callus (i.e. endosteal, periosteal and intercortical). The tissue fractions are calculated by the following procedure: first the spatial images are binarized using tissue-specific thresholds (0 means that the tissue is not present, 1 means that the tissue is present in a grid cell). Subsequently, an equal weight is assigned to the different tissues, i.e. if a grid cell contains three tissues, the area of that grid cell is divided by three in the final calculations of the tissue (area) fractions [23].

## Results

### *In silico* and *in vivo* non-union model

**Figure 4:**
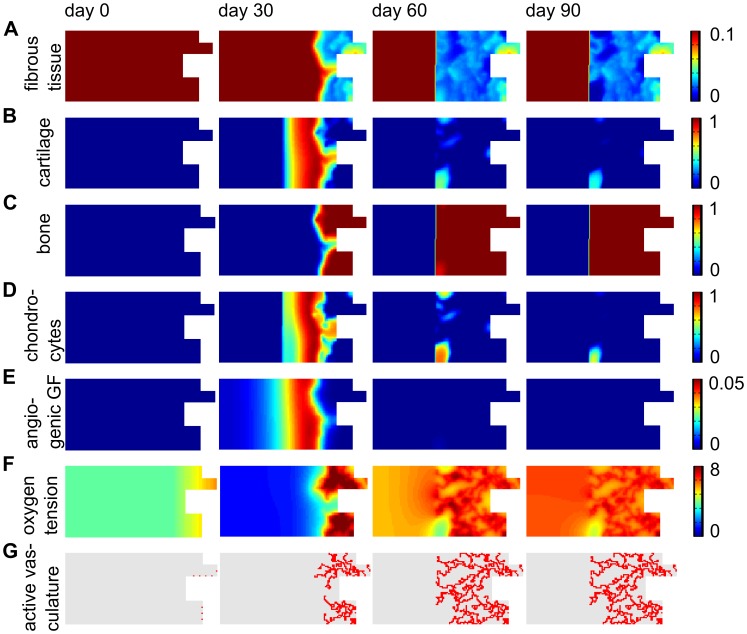
The predicted spatiotemporal evolution of fracture healing in a critical sized defect (5 mm). (A) fibrous tissue density (×0.1 g/ml), (B) cartilage matrix density (×0.1 g/ml), (C) bone matrix density (×0.1 g/ml), (D) chondrocyte density (×10^6^ cells/ml), (E) angiogenic growth factor (GF) concentration (×100 ng/ml), (F) oxygen tension (×1%) and (G) active vasculature.

**Figure 5:**
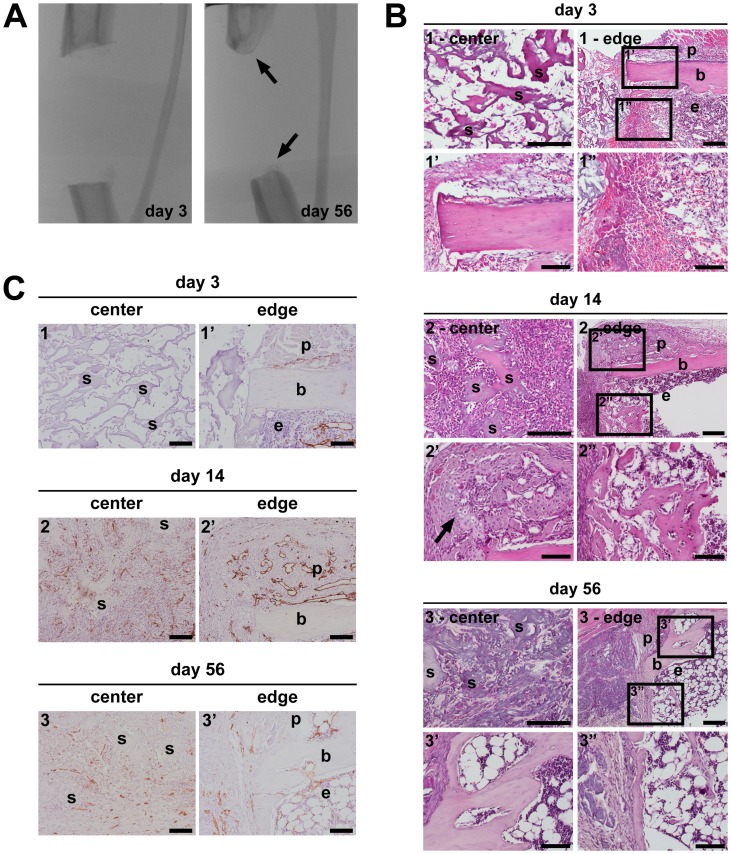
Analysis of healing of large bone defects in the tibia of mice in which a collagen scaffold seeded with periosteal cells was implanted. (A) Radiographic images of the treated defects (5 mm gap size) showing capping (arrows) of the cortical bone ends, but absence of full defect healing over the course of 56 days. (B–C) Images of H&E (B) and CD31 (C) stained histological sections of treated bone defects obtained at 3, 14 or 56 days after surgery. H&E provides a general image of the tissues under investigation whereas CD31 specifically stains the blood vessels. The arrow in B2′ indicates cartilage remnants adjacent to new bone tissue. Scale bars: 200 µm and 100 µm (′,″)in B, 100 µm in C. b: cortical bone, p: periosteal region, e: endosteal region, s: scaffold.

A qualitatively similar healing progression is predicted by the simulation results as observed experimentally (Figures 4 and 5). At early time points a periosteal reaction, characterized by a thickening of the periosteal layer (Figures 5-B1, B1′ and C1) as well as the presence of a hematoma, a fibrous-like tissue associated with the presence of numerous (red) blood cells (Figures 5-B1, B1″), are observed at the cortical host bone site, both supporting the initial and boundary conditions that were applied in the multiscale model (Figure 3-B). In the center of the large bone defect no signs of tissues or infiltration of blood vessels are detected, only scaffold material together with a low cellularity is observed (Figures 5-B1-center, C1′), corresponding to the predictions of the *in silico* model (Figure 4-G).

On day 14, a periosteal endochondral ossification reaction is seen, evidenced by the presence of cartilage (large round cells staining grey-blue with H&E) and trabecular-like bone (dense matrix, staining bright pink with H&E, with the clear presence of embedded osteocytes) (Figures 5-B2, B2′; arrow indicates cartilage), while direct bone formation occurs endosteally (Figures 5-B2, B2″). The mathematical model predicts a similar distribution of tissue formation, i.e. direct bone formation near the bony ends and endochondral ossification further away in the fracture callus (Figure 4-B,C). In the center of the defect only a highly dense fibrous tissue is observed in both the experimental, the scaffold remains stained pink-blue with H&E but lack the presence of embedded cells (Figure 5-B2-center), as well as the mathematical model (Figure 4-A). In contrast to the experimental model, the mathematical model does not predict any blood vessels in the central callus area (indicated with dots in Figure 3). These vessels, however, appear to be small and immature whereas the blood vessels that are associated with the sites of bone formation are large and mature (compare Figures 5-C2 and C2′). This discrepancy might be explained by the fact that the mathematical framework only models angiogenesis, i.e. blood vessel growth through the creation of new vessel branches from existing ones, whereas vasculogenesis, i.e. *de novo* network formation from scattered endothelial cells, is not included here. Indeed, after bone fracture the hematoma will be filled with blood, containing amongst others endothelial precursor cells, which could explain the small, immature blood vessels observed experimentally. We would like to stress, however, that this is a first hypothesis that is currently being explored further.

Notice the closure of the bone marrow canal by new bone on day 56, separating the bone marrow (right) from the scaffold region (left) (Figure 5-B3, B3′, B3″). As such, capping of the bone ends has occurred both in the experimental and the mathematical model (Figure 4-C). The blood vessels in the center are still much smaller compared to those near the edges of the defect (compare Figure 5-C3 and C3′). In the center of the defect no signs of bone formation are detected, only fibrous tissue is seen, at this time point associated with a very low cellular content (Figure 5-B3-center). Also in the mathematical model no additional bone formation is predicted between post fracture day (PFD) 60 and 90, thereby classifying this fracture as a non-union [1], [2].

After this qualitative validation of the model predictions with the experimental observations of bone healing in a large defect, the model was used to understand the mechanisms underlying the occurrence of fracture non-unions. It appears that in the mathematical model, chondrogenic differentiation and cell survival are severely impaired in the central callus area (indicated with dots in Figure 3) due to the harsh hypoxic conditions (optimal oxygen tension for chondrogenic differentiation is 3%, minimal oxygen tension for MSC and chondrocyte survival is 0.5%, see Figure 2) (Figure 4-D,F). Consequently, the angiogenic growth factor (*g_v_*), which is the major stimulus for vascular growth and as such endochondral ossification, is not produced in the central callus area (Figure 4-E). As a result, the bone healing stops after capping of the bony ends, resulting in an atrophic non-union (Figure 4-C). Note that the predicted bone front extents further into the callus than observed in the *in vivo* model. This might be due to some limitations of the computational model. Firstly, in the current model all the progenitor cells can differentiate towards both the chondrogenic and osteogenic lineage, depending on the local growth factor concentrations and oxygen tensions. In reality, however, it has been shown that the progenitors from the endosteal callus can only differentiate towards the osteogenic lineage, resulting in the absence of cartilage in the endosteal callus [28]. Progenitor cells from the periosteum do have the capability to differentiate to both lineages, explaining why endochondral ossification mainly occurs in the periosteal callus [28]. As such, the current simplification of the model leads to an overestimation of the amount and the location of the cartilage matrix, resulting in an overestimation of the predicted bone formation. Secondly, the current model does not account for changes in callus size and shape during the healing process which may also influence the bone formation process.

### The defect size of murine bone fractures becomes critical at 3 mm

**Figure 6:**
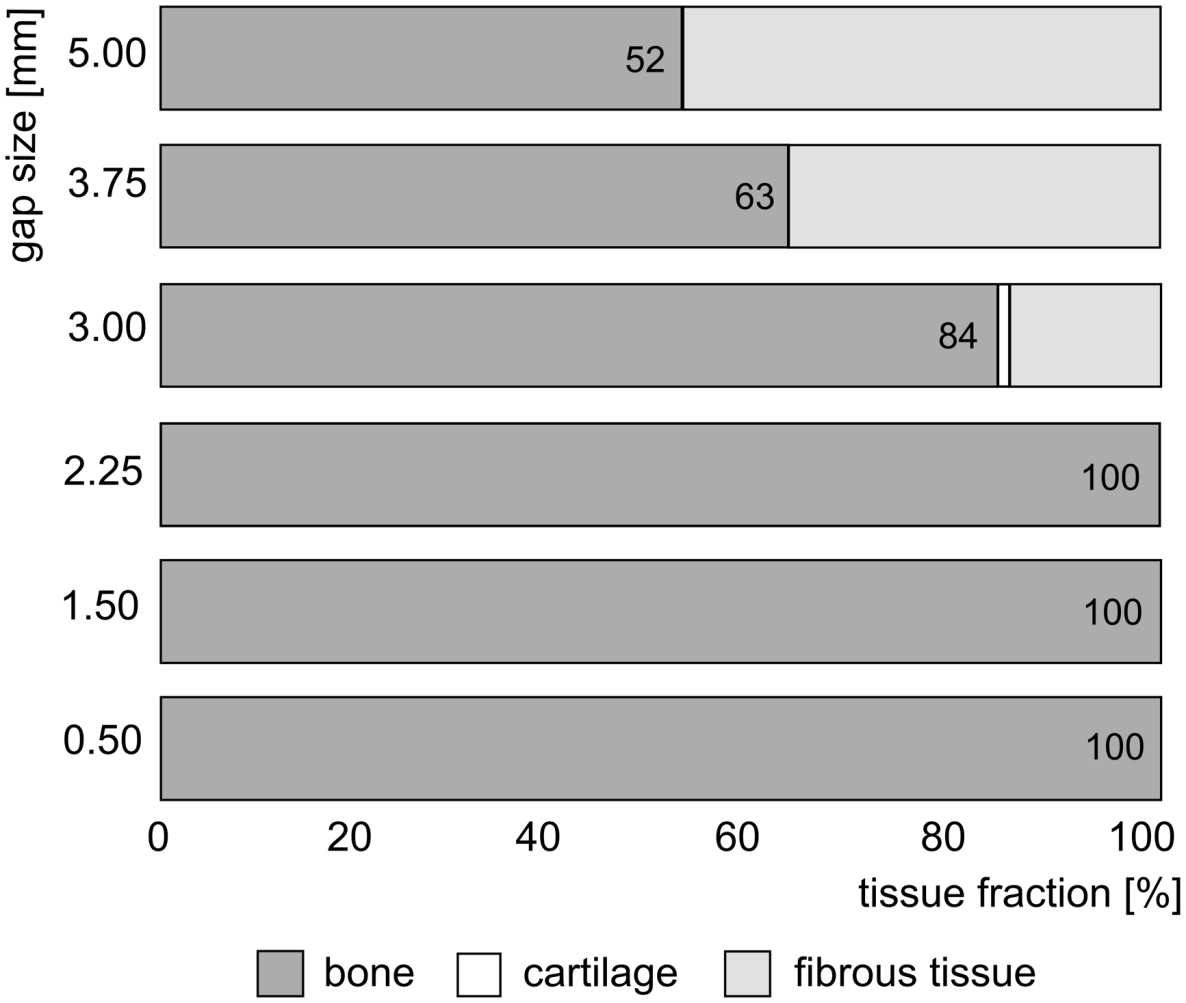
Predicted tissue fractions at post fracture day (PFD) 90 in bone defects of varying sizes. The 5 mm defect size will be further investigated in the remaining part of this study. The femurs at the right hand side of the figure schematically represent the bone healing outcome at PFD 90, i.e. the formation of a union or a non-union.

After establishing the *in silico* and *in vivo* non-union model, the *in silico* model was further used to explore the influence of the gap size on the healing outcome (Figure 6). By increasing the gap size, the bone tissue fraction at PFD 90 is reduced whereas the cartilage fraction remains similar (close to zero) and the fibrous tissue fraction is greatly increased (Figure 6). Although the bone tissue fraction reaches 84% in a 3 mm defect, there is no cortical bridging which indicates the formation of a non-union. The simulation therefore predicts that a murine bone defect becomes critical at 3 mm. In the remaining part of this study we will focus on the bone regeneration process in 5 mm defects, in correspondence with the *in vivo* set-up described above.

### The biological potential of the fracture callus is necessary but insufficient for complete bone healing

Since for all the different gap sizes explored in Figure 6, the same set of initial and boundary conditions was employed, the occurrence of fracture non-unions might be attributed to an inadequate vascularization of the central callus region. More specifically, the ingrowing vasculature which originates from the bony ends, needs to cover a larger distance in larger defects, resulting in a too late vascularization of the central fracture area (Figure 4-G) and consequently harsh hypoxic conditions (Figure 4-F). As was explained above, these hypoxic conditions lead to cell death thereby arresting the production of angiogenic growth factors and ultimately the bone healing process (Figure 4-C). Clearly, the spatiotemporal patterns of oxygen tension are an important determinant of successful bone repair which prompted us to investigate the complex interplay between oxygen delivery, diffusion and consumption in a critical size defect (5 mm). An extensive sensitivity analysis was performed on the parameter values describing the delivery of oxygen (*G_n_*), the diffusion of oxygen (*D_n_*) and the oxygen consumption by osteoblasts (*Q_b_*), chondrocytes (*Q_c_*), MSCs (*Q_m_*) and fibroblasts (*Q_f_*). Moreover, since experimental evidence has shown that the biological potential (e.g. the amount of osteoprogenitor cells and growth factors present) might be greatly reduced in critical size defects [26], [27], we also explored the influence of the initial conditions (*c_m,init_, g_bc,init_, c_f,init_, m_f,init_, n_init_*) in the central callus area (indicated with dots in Figure 3) on the fracture healing outcome (Table S1 in the supplementary material).

The initial position of the endothelial cells (see Figure S1 in the supplementary material), has a small influence on the final bone tissue fraction (+/−2%). This difference can be attributed to a different spatial filling of the blood vessels in the 2D simulated geometry and is in the same range as the influence of the stochastic component in the description of blood vessel migration on the simulation outcome (+/−3%) [24]. Based on these findings, we consider deviations of more than 2% with respect to 50% of bone tissue fraction to be sufficient to warrant further analysis. In order to gain more understanding in the complex non-linear dynamics of the oxygen model, the mechanisms underlying these significant deviations were investigated and are discussed in more detail below.

The sensitivity analysis revealed a non-linear influence of the initial amount of MSCs (*c_m,init_*) on the bone tissue fraction at PFD 90. This can be explained by the fact that on the one hand a low initial concentration of MSCs (*c_m,init_*<2.10^4^ cells/ml) reduces the biological potential of the fracture site since less cells can contribute to the bone healing process. On the other hand, a high initial concentration of MSCs (*c_m,init_*>2.10^5^ cells/ml) will worsen the detrimental hypoxic conditions in the central callus region due to the increased amount of oxygen consumption. The initial concentration of fibroblasts (*c_f,init_*) does not show this non-linear behavior. High initial concentrations of fibroblasts and/or MSCs are detrimental (*c_f,init_*>5.10^5^ cells/ml) since the increased oxygen consumption will lower the average oxygen tension in the central callus area. Contrary to the MSCs, low initial concentrations of fibroblasts do not seem to have a major influence on the final amount of bone formation. This is mainly because fibroblasts do not contribute to the biological potential of the hematoma as they cannot differentiate towards the osteogenic or chondrogenic lineage.

The sensitivity analysis also indicates that the amount of osteochondrogenic growth factors present in the fracture hematoma (*g_bc,init_*) is a critical determinant of the final amount of bone formation. Indeed, increasing the growth factor concentration results in a significant increase in the amount of bone formation measured after 90 days of healing. This result can be attributed to an increased chondrogenic differentiation which limits the oxygen consumption since chondrocytes consume less oxygen than MSCs. As such, the central hypoxic area will be reduced leading to more bone formation.

After the inflammation phase, the fracture callus is filled with granulation tissue (represented here by *m_f,init_*). It appears that a large amount of granulation tissue negatively influences the fracture healing outcome which is due to its inhibitory effect at large matrix densities on the proliferative capacities of MSCs, fibroblasts, chondrocytes and osteoblasts.

Similar to the initial amount of MSCs also the initial oxygen tension (*n_init_*) has a non-linear effect on the final amount of bone formation. Very low oxygen tensions (*n_init_*<0.5%) lead to a larger hypoxic area and less bone formation whereas oxygen tensions above 4% (*n_init_*>4%) hamper the proliferation of chondrocytes, thereby disrupting the cartilage production and consequently the endochondral ossification process. Interestingly, in the intermediate range of oxygen tensions (0.5%<*n_init_*<4%), lower initial oxygen tensions appear to result in more bone formation (Table S1, 0.7% versus 3.7% oxygen tension of the standard compromised condition). Although intuitively we would expect that these low oxygen tensions would lead to worse hypoxic conditions, model analyses show that the average oxygen tension in the fracture callus remains above 0.8% during the entire healing period (note that the low oxygen tensions of the central callus area are averaged with the high oxygen tensions near the bony ends), which is well above the oxygen threshold for chondrocyte and MSC cell death (i.e. 0.5%). As such the oxygen tension is low enough to inhibit extensive proliferation (as the chondrocytes and MSCs preferentially proliferate at 3% and 4% oxygen tension respectively, Figure 2) and therefore avoiding too much oxygen consumption, but high enough to keep a small amount of remaining stem cells alive. Moreover, the oxygen consumption is not only reduced due to the smaller amount of consuming cells. The cellular consumption of oxygen is also oxygen dependent, leading to a lower cellular consumption in low oxygen environments. It is the combination of these effects that limits the drop of the average oxygen tension, allowing the MSCs to survive and contribute to the bone healing process for a longer period of time (40 days for case *n_init_* = 0.7% versus 4 days in the standard condition). A similar reasoning can be made for the case where an initial gradient of oxygen tensions was applied to the central callus region (*n_init,gr_* = 0.8%/mm*x). In this simulation the oxygen tension varied from 0% in the middle of the callus to 4% at the bony ends. The low oxygen tensions in the central area supported the maintenance of a small population of MSCs for a longer period of time (6 days versus 4 days in the standard condition). This resulted in a larger amount of cartilage and finally bone. Note that this specific gradient in oxygen tension is less beneficial for the amount of bone formation at PFD 90 than a uniform distribution of 0.7%, as in the case of the gradient the oxygen tension in the middle of the callus is too low to sustain cell viability.

Besides investigating the influence of the initial conditions, the sensitivity analysis also focused on the complex interplay between oxygen delivery (*G_n_*), diffusion (*D_n_*) and consumption (*Q_b_, Q_c_, Q_m_, Q_f_*). Altering the oxygen delivery (*G_n_*) by the vasculature has a large effect on the final amount of bone formation. Very low values of oxygen delivery increase cell death in the central hypoxic area, resulting in the absence of any bone formation. Increasing the value of oxygen delivery slightly improves the fracture healing outcome. Note that, although the bone tissue fraction is 37% in case of *G_n_* = 22.10^−12^ mol/cell.day and 55% in case of *G_n_* = 3.2.10^−12^ mol/cell.day, the spatial extent of bone ingrowth at PFD 90 is very similar (results not shown). This is however masked by the increased proliferation and matrix production of fibroblasts who thrive in the well-oxygenated environment created by *G_n_* = 22.10^−12^ mol/cell.day. As such, the bone tissue fraction for *G_n_* = 22.10^−12^ mol/cell.day is reduced with respect to *G_n_* = 3.2.10^−12^ mol/cell.day. The parameter values of the cell-specific oxygen consumption rates (*Q_b_, Q_c_, Q_m_, Q_f_*) also influence the outcome of the model significantly. For all cell types, it is beneficial to reduce the oxygen consumption rates since this will limit the decrease in oxygen tension in the central fracture area and consequently the amount of cell death. This benefit is greatest for the MSCs and chondrocytes as these cell types mainly populate the central fracture area and contribute to the hypoxic conditions encountered here. Conversely, the amount of bone formation is greatly reduced when the oxygen consumption rate of the MSCs (*Q_m_*) or chondrocytes (*Q_c_*) is increased. The model outcome is also negatively affected by a high osteoblastic oxygen consumption rate (*Q_b_*) whereas a high fibroblastic consumption rate (*Q_f_*) only slightly reduces the final amount of bone. In the first case, the oxygen tension near the bony ends is reduced, resulting in hampered osteogenic differentiation and limited bone formation. In the latter case, the fibroblasts reduce the oxygen tension in the entire callus area (the fibroblasts are initially uniformly distributed in the fracture callus) but this drop is limited due to the small amount of fibroblasts present. Interestingly, a similar reasoning does not hold for the MSCs (although they are also initially uniformly distributed and limited in cell population) since they mainly grow in the central fracture zone whereas the fibroblasts optimally proliferate in a well-oxygenated environment such as the tissues surrounding the bony ends. As such, a high oxygen consumption rate of MSCs severely impairs the bone formation process whereas a high oxygen consumption rate of fibroblasts only slightly reduces the amount of bone formed at PFD 90.

It can be noticed from Table S1 and Figure S2 that the diffusion properties of oxygen have a major impact on the simulation outcome. Reducing the diffusion coefficient of oxygen impairs the bone formation due to the creation of a larger hypoxic zone (Figure S2-A,C). Increasing the diffusion coefficient appears to be beneficial although a closer look at these simulation results reveals that the endochondral process is not captured correctly anymore with bone formation largely preceding the ingrowth of new blood vessels (Figure S2-D,F). Note that also in this case a non-union is formed, since there is no cortical bridging, even though a bone tissue fraction of 89% is reached (Table S1). Increasing the diffusion coefficient even further results in a complete absence of bone formation since the resulting oxygen tensions are too low for any cell type to survive (Figure S2-H) (see Supporting Text S3).

In conclusion, we can state that the initial conditions have an important impact on the final amount of bone formation. They are however not sufficient to result in complete healing of critical size defects due to insufficient vascularization of the central callus area, leading to hypoxic conditions and cell death. As such, an adequate and timely restoration of the vasculature appears to be an important determinant of the healing outcome.

### Contribution of the muscle vasculature to the vascularization of the fracture callus is beneficial for bone healing

Inspired by the importance of a timely vascularization as well as by the limited biological potential of the fracture hematoma, we explored the influence of the host environment on the bone healing process in critical size defects. It appears that the fracture healing process is intimately linked to the surrounding muscle envelope since clinical evidence has found that open fractures with significant muscle injury complicate fracture healing and are a risk factor for the development of non-unions [5]–[7]. Moreover, tibial shaft fractures, which are only covered by a thin layer of soft tissue, are prone to a number of complications often resulting in additional surgical interventions [5], [9], [29]. There are a number of ways by which the skeletal muscle can contribute to the bone healing process. Firstly, experimental studies have shown that blood vessels originating in the overlying muscle contribute to the vascularization of the fracture callus [30], [31]. Secondly, muscle cells are a source of growth factors (e.g. FGF-2, TGF-β) [32] as well as progenitor cells [33]–[35]. Thirdly, the muscle envelope might provide the adequate biomechanical stimuli required for successful bone healing [36], [37].

**Figure 7:**
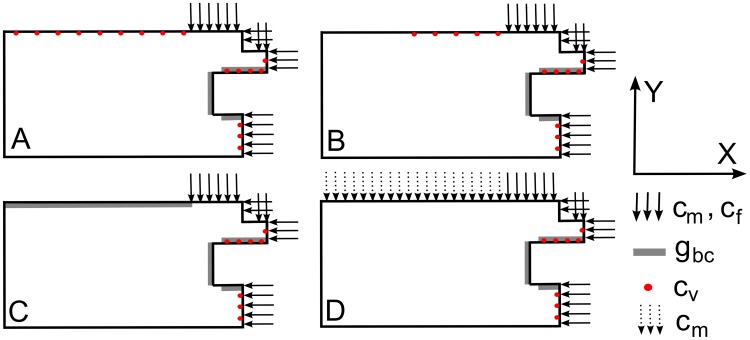
Schematic representation of the different boundary conditions that were applied to the critical size defect. (A) full vascular contribution of the overlying muscle (case A, Figure 8) (B) partial vascular contribution of the overlying muscle (case B, Figure 8) (C) Dirichlet boundary condition of osteochondrogenic growth factors (case C, Figure 8) (D) Dirichlet boundary condition of MSCs (case D, Figure 8). The origin of the coordinate system is placed in the left bottom corner of the geometrical domain.

**Figure 8:**
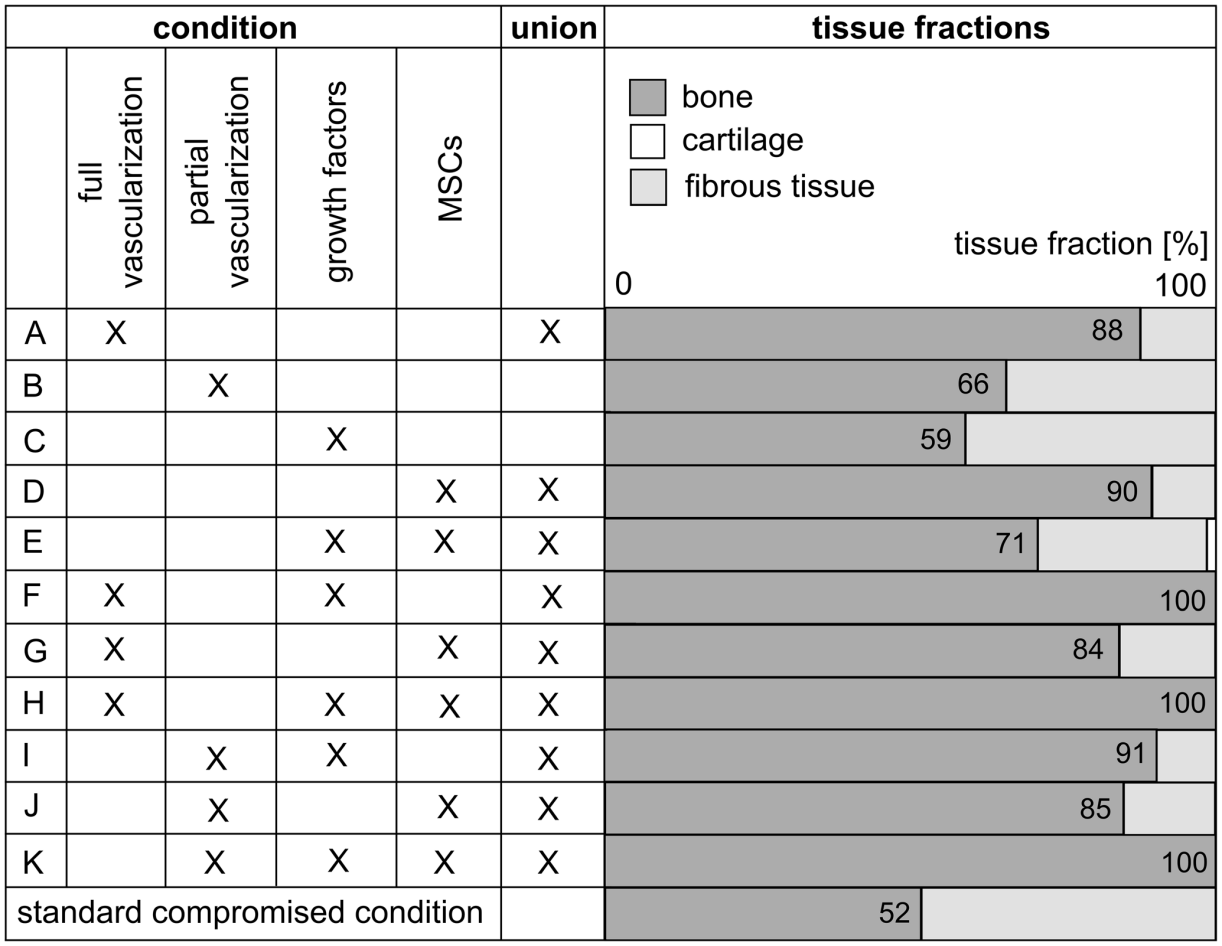
Influence of the muscle as a source for vascularization, MSCs, growth factors or a combination thereof on the bone regeneration process. The tissue fractions are measured at post fracture day (PFD) 90. The standard condition is indicated in bold and has the following dimensionalized parameter values for the initial conditions in the central area of the fracture callus: *c_m,init_* = 2.10^3^ cells/ml, *g_bc,init_* = 10 ng/ml, *c_f,init_* = 1.10^4^ cells/ml, *m_f,init_* = 0.01 g/ml, *n_init_* = 3.7%. Table S2 summarizes the tissue fractions quantitatively.

In order to further unravel the potential mechanisms of interaction that exist between the bone regeneration process and the overlying skeletal muscle, the role of the skeletal muscle as a source for vascularization, progenitor cells and growth factors or a combination thereof was investigated by applying different boundary conditions to the *in silico* model (Figure 7–8). More specifically, the contribution of the muscle to the vascularization of the fracture callus was simulated by initializing additional endothelial cells on the border of the central callus area with the muscle, either partially or fully covering the fracture gap. The influence of the muscle as a source of MSCs or growth factors was represented by a Dirichlet boundary condition, applied to the upper border for the entire duration of the simulation (i.e. 90 days) and fully covering the fracture gap (Figure 7). The value of the Dirichlet boundary conditions is equal to the ones applied in the standard case, i.e. 2.10^4^ cells/ml for the MSCs and 2 µg/ml for the osteochondrogenic growth factors [25], [38]–[40]. Since the mathematical model does not take into account any mechanoregulatory stimuli, the influence of mechanoregulatory stimuli generated by the overlying muscle on the bone formation processes cannot be evaluated in this study.

The results, summarized in Figure 8, underline the importance of the host environment for successful fracture healing since all the investigated conditions improve the amount of bone formation with respect to the standard condition or result even in bridging of the critical size defect. Note that the host environment is also more efficient in stimulating the bone regeneration process than the initial conditions tested in Table S1, since the host environment continuously provides the fracture callus with fresh growth factors, cells and blood vessels (or a combination thereof) whereas the initial conditions represent only a single (initial) contribution to the bone regeneration process. In order to limit the length of the paper, we will touch upon the most important findings of Figure 8 and refer the reader to Supporting Text S4 for an in depth discussion of the results. Both the contribution of the muscle as a source of vascularization (case A) as well as of osteoprogenitor cells (case D) results in the formation of a union, whereas a partial supply of blood vessels from the host environment (case B) or muscle derived release of osteochondrogenic growth factors fails to result in a complete bridging of the defect (Figure S3). In most cases, except for cases E and G, the combination of two or more boundary conditions enhances the bone formation process. Indeed, the combined delivery of cells and growth factors results in less bone formation (case E) than the delivery of cells alone (case D). Figure 8 also shows that without vascular ingrowth from the muscular environment, the delivery of cells results in the largest amount of bone formation (case C versus case D). However, if the fracture callus is fully or partially vascularized by the overlying muscle, the delivery of growth factors is more beneficial than the delivery of cells for the final healing outcome (case F versus G, case I versus J).

In conclusion, we can state that the contribution of the host environment, and more specifically its role as a source of vascularization is critical for successful bone healing. Interestingly, the results indicate that the lack of adequate vascularization can be rescued by a continuous delivery of osteoprogenitor cells, potentially in combination with osteochondrogenic growth factors.

### Treatment strategies for critical size defects in a permissive host environment

**Figure 9:**
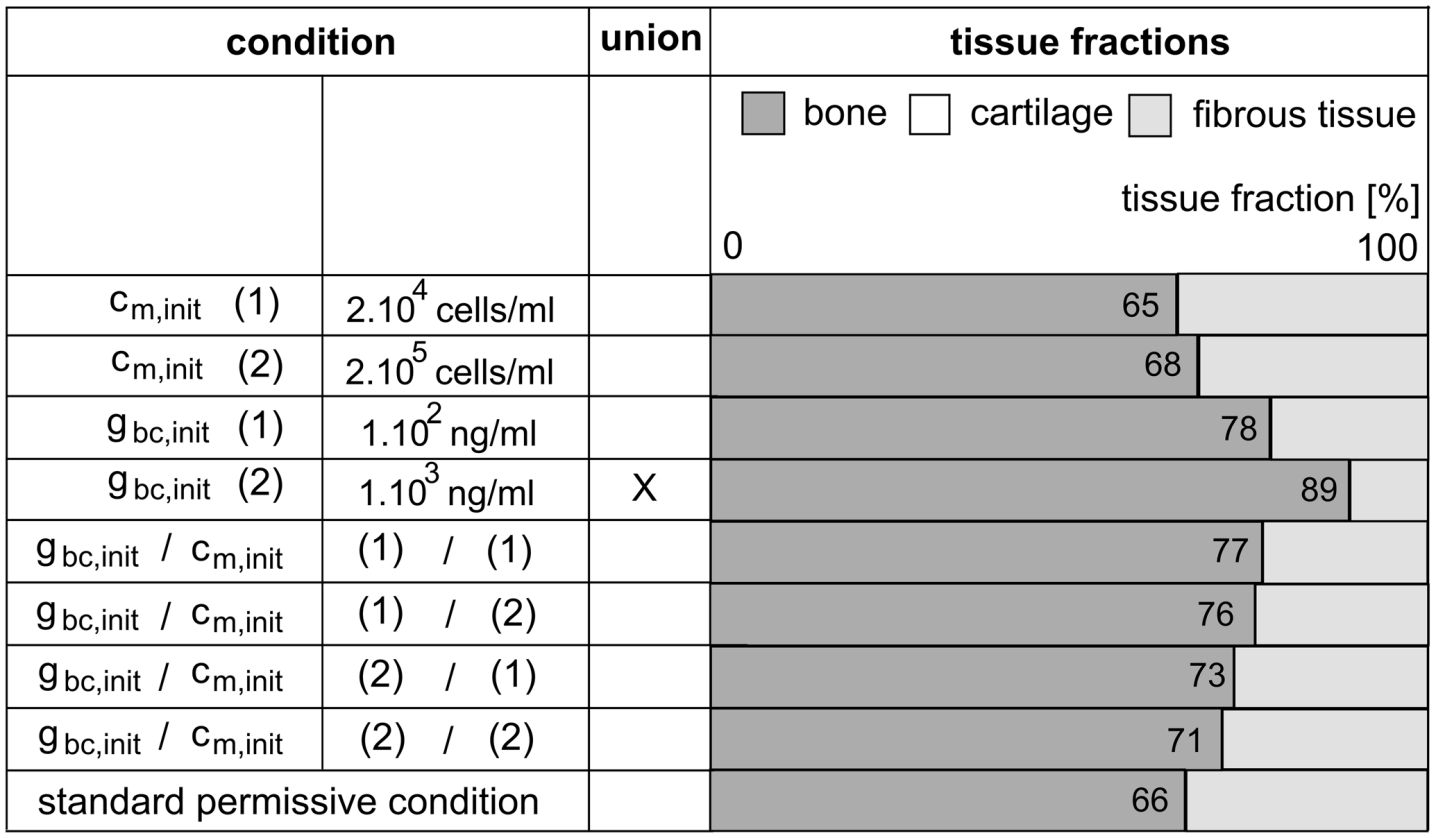
Results of three types of treatment strategies in a permissive host environment where the overlying muscle partially contributes to the vasculature of the fracture callus. The tissue fractions are measured at PFD 90. The standard condition is indicated in bold and has the following dimensionalized parameter values for the initial conditions in the central area of the callus: *c_m,init_* = 2.10^3^ cells/ml, *g_bc,init_* = 10 ng/ml, *c_f,init_* = 1.10^4^ cells/ml, *m_f,init_* = 0.01 g/ml, *n_init_* = 3.7%. Table S3 summarizes the tissue fractions quantitatively.

Intrigued by the results of the previous section, we wondered if the lack of adequate vascularization could also be rescued by a single contribution of (more optimal) initial conditions. Or, from another perspective, whether the initial conditions that were insufficient to result in successful bone healing in a compromised environment (Table S1), would be able to stimulate the bone regeneration process more in a permissive host environment. In order to answer this question, we use the model in which the fracture callus is partially supplied by blood vessels from the overlying muscle (case B, Figure S3) since this environment is not as compromised as the standard compromised condition (Table S1) but nevertheless results in the formation of a non-union without additional cells or growth factors (Figure 8, case B). We tested three potential treatment strategies: the injection of growth factors, the injection of cells and the injection of a combination product. All the injections take place at day zero, making them initial conditions (Figure 9).

According to the results of Figure 9, all the treatment strategies yield at least the same (within 2% of intrinsic variability) or more bone formation than a non-treated fracture in a permissive environment. Moreover, the permissive environment is clearly beneficial since the amount of bone formation is increased with respect to the compromised environment for all the treatment conditions (Table S1).

**Figure 10:**
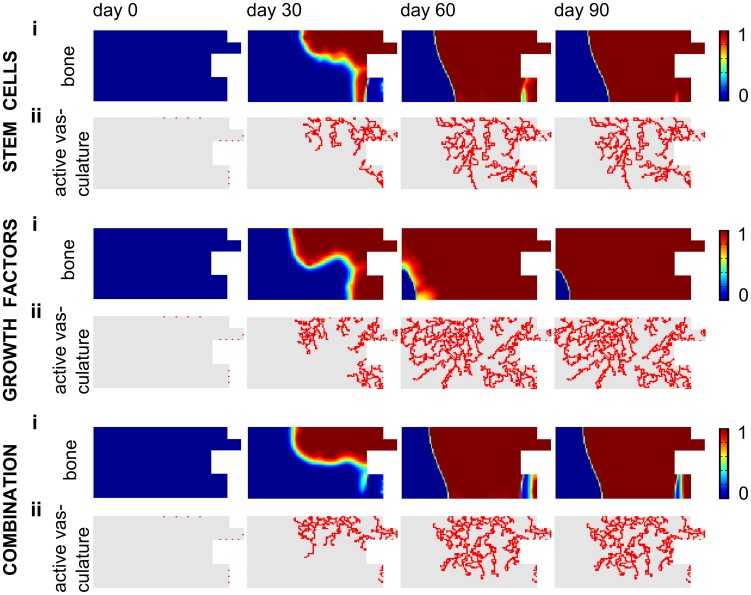
The predicted spatiotemporal evolution of fracture healing in a permissive environment after different treatments. (i) bone matrix density (×0.1 g/ml), (ii) active vasculature during fracture healing in a permissive environment after an injection of osteoprogenitor cells (*c_m,init_* = 2.10^5^ cells/ml), growth factors (*g_bc,init_* = 1.10^3^ ng/ml) or a combination thereof (Figure 9).

**Figure 11:**
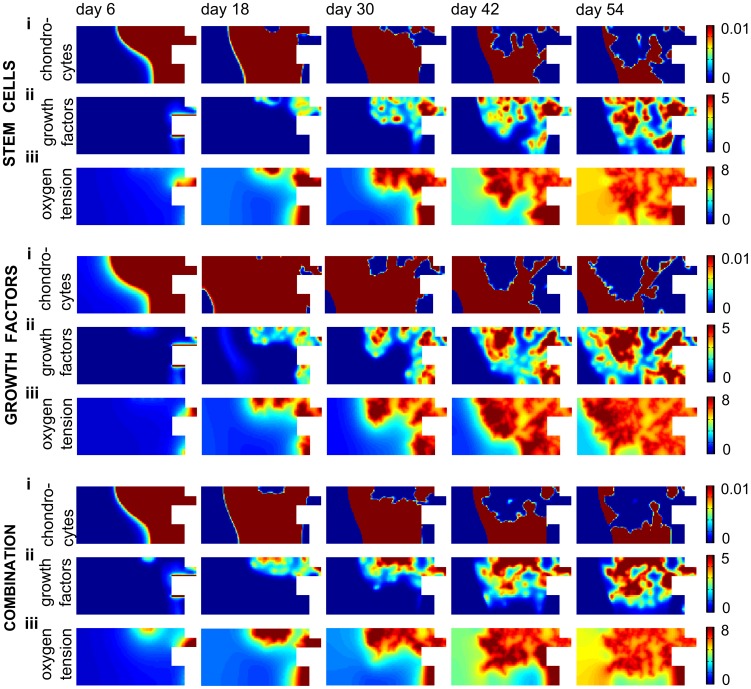
The predicted spatiotemporal evolution of fracture healing in a permissive environment after different treatments. (i) chondrocyte concentration (×10^6^ cells/ml), (ii) growth factor concentration (×100 ng/ml) and (iii) oxygen tension (×1%) during fracture healing in a permissive environment after an injection of osteoprogenitor cells (*c_m,init_* = 2.10^5^ cells/ml), growth factors (*g_bc,init_* = 1.10^3^ ng/ml) or a combination thereof (Figure 9). Note that the scale of the chondrocyte concentration is different from the one displayed in Figure 4.

The injection of precursor cells does not significantly improve the bone healing outcome since the vascularization of the central callus area is still delayed, resulting in hypoxic conditions and cell death (Figure 11). The injection of osteochondrogenic growth factors is able to heal the critical size defect surrounded by a muscular envelope that partially contributes to its vascularization if the concentration is sufficiently high (Figures 9–10–11). The mechanism of action underlying this result can be explained as follows. The large initial concentration of growth factors will lead to the differentiation of the osteoprogenitor cells into chondrocytes, which consume less oxygen than MSCs. Consequently, the pool of oxygen-consuming MSCs is reduced thereby limiting the oxygen consumption. As such, a large initial concentration of growth factors makes the hypoxic area shrink, finally leading to the successful healing of the critical size bone defect (Figure 11).

The injection of the combination product has improved the amount of bone formation but is not as beneficial as osteochondrogenic growth factor injections alone (Figures 9–10–11). Indeed, the growth factor concentration is not high enough to commit the entire population of MSCs to the chondrogenic lineage. As such, a small amount of MSCs remains undifferentiated and can continue to proliferate and consume oxygen. Since MSCs consume more oxygen than chondrocytes, the remaining MSC pool increases the drop in oxygen tension and consequently cell death. As a result, the amount of bone formation is lower than in the case of growth factor treatment alone.

**Figure 12:**
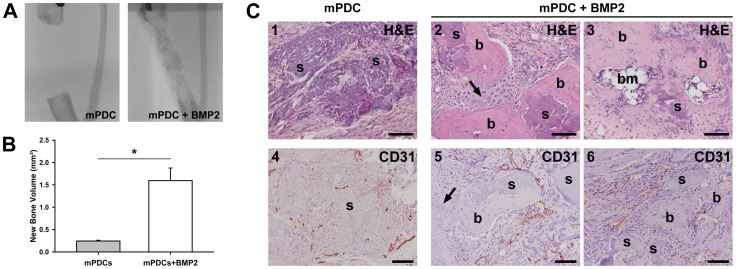
Implantation of periosteal cells overexpressing the osteochondrogenic growth factor BMP-2 promotes healing of large bone defects in mice. (A–B) Radiographic analysis (A) and μCT-based quantification (B) of bone formation in large bone defects treated with collagen scaffolds containing periosteal cells (mPDC; n = 3) or periosteal cells overexpressing BMP2 (mPDC+BMP2; n = 5) showing the clear positive effect of the presence of BMP2 on bone healing (*p<0.05). (C) Histological sections of defects treated with mPDC or mPDC+BMP2 at day 56 after surgery, H&E (1–3) and CD31 (4–6) stainings are shown. All images were taken in the central region of the defect. Arrows indicate small regions of cartilage adjacent to the newly formed bone. Scale bars: 100 µm. b: bone, bm: bone marrow, s: scaffold.

The predictions of the mathematical model are compared with the results of the *in vivo* set-up where the influence of BMP-2 overexpressing periosteal cells on bone formation in large defects was explored. Defects, treated with a collagen scaffold containing mPDCs, thereby mimicking the initial conditions of the computational model, show little bone formation (Figure 12-B), which is also predicted by the mathematical model (Figure 9, standard permissive condition). Interestingly, while defects treated with mPDCs show no presence of bone or cartilage in the center of the defect (Figure 12-C1), large amounts of bone and the presence of cartilage (arrow) and bone marrow are noted in the defects treated with BMP-2 overexpressing mPDCs (Figure 12-C2, C3). Clearly, the presence of BMP-2 enhances the bone formation process which results in a clinical union. Similarly, the computational model predicts that the injection of only growth factors is sufficient to heal a large defect in a permissive environment (Figure 9, *g_bc,init_*). Note, however, that in the experimental set-up BMP-2 overexpressing cells are implanted whereas computationally an initial bolus injection of growth factors is simulated.

In the experimental model, the sites of bone formation are closely associated with numerous large blood vessels (indicated in dark brown by the CD31 staining, Figure 12-C5, C6), in contrast to the small blood vessels observed in the center of the defects treated with mPDCs only (Figure 12-C4). In the mathematical model, the blood vessel formation is also closely connected to the bone formation process (Figure 10). In the central callus area we hypothesize that the small blood vessels observed *in vivo* arise through vasculogenesis, which is not accounted for in the mathematical model. However, since these small blood vessels appear to be immature and not associated with bone formation, the mathematical model does predict the correct tissue distribution in the central callus area even in the predicted absence of small blood vessels. As expected, no blood vessels are observed at the site of cartilage formation (Figure 12-C5, arrow).

While the influence of the amount of seeded cells alone or in combination with BMP-2 overexpression was not explored experimentally, the computational simulations predicted an improvement in the amount of bone formation but not a complete healing of a large bone defect (Figure 9, *c_m,init_* and *c_m,init_*/*g_bc,init_*).

Note that in a compromised environment, a large defect will develop into a non-union, irrespective of a growth factor treatment (Table S1, standard compromised condition), whereas in an environment with full vascular ingrowth from the overlying muscle, a union will develop, irrespective of a growth factor treatment (Figure 8, case A). As such, since the computational predictions of the growth factor treatment in a permissive environment reproduce the *in vivo* observations correctly, one may speculate that the muscle overlying the large defect in the *in vivo* set-up partially contributed to the vascularization of the fracture callus and consequently the bone healing process. Further characterization of the origin of the vasculature growing towards the defect area would be required to confirm this.

From the results discussed above, we can conclude that a single injection of osteochondrogenic growth factors is able to compensate for the lack of adequate vascularization. Since a single injection of cells fails to promote complete bridging of the critical size defect in a permissive environment, a sequence of cellular injections might be more appropriate strategy.

### Treatment strategies for critical size defects in a compromised host environment

**Figure 13:**
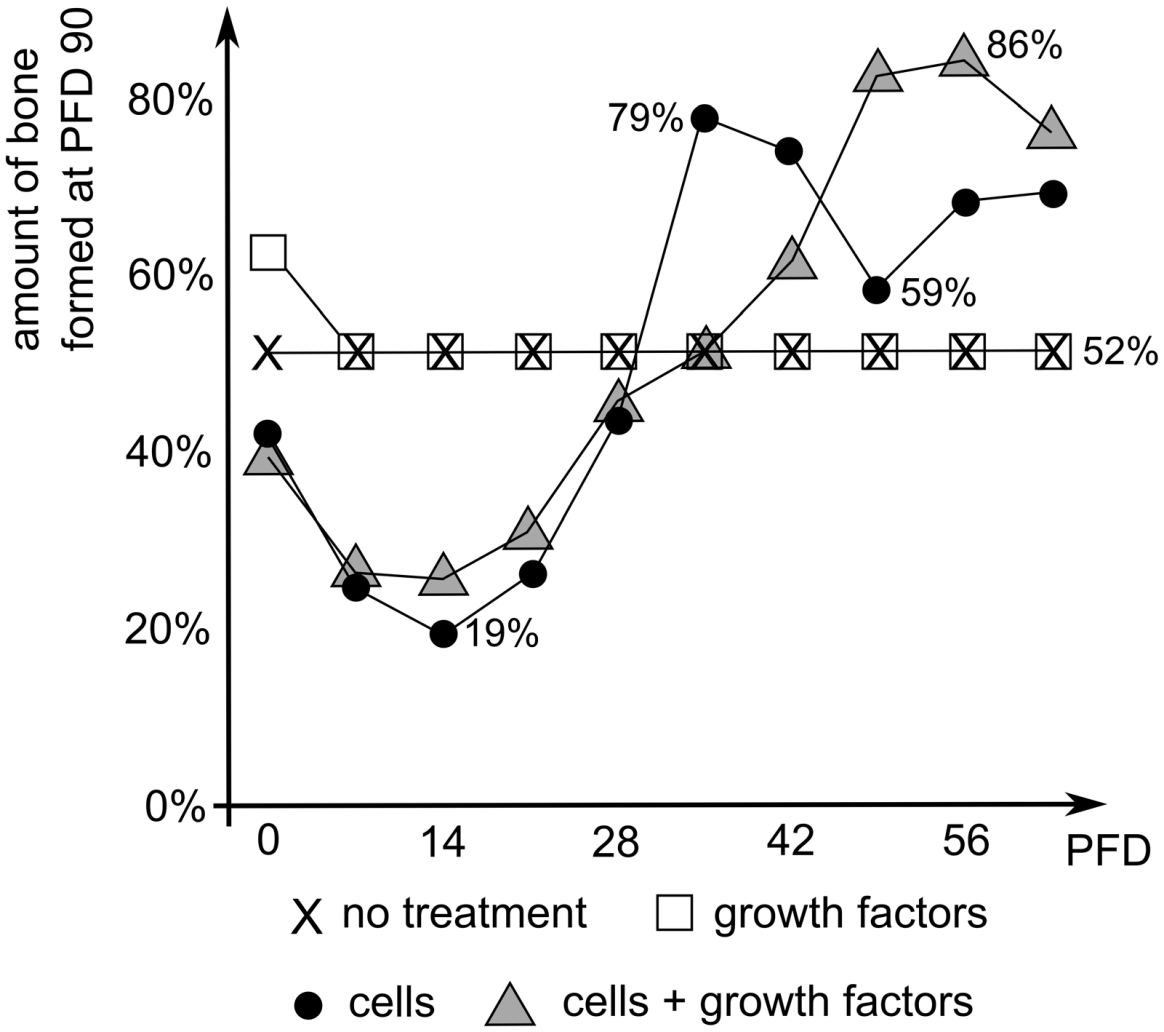
Predicted amount of bone formation at PFD 90 in a large defect surrounded by a compromised environment as a function of the PFD at which the treatment was initiated. The treatment consists of a single injection of cells (*c_m_* = 1.10^6^ cells/ml), growth factors (*g_bc_* = 1.10^3^ ng/ml) or a combination thereof. The results of injections at PFD 0 can also be found in Table S1.

Finally, we used the *in silico* model to optimize the treatment strategy of the previous section for critical size defects surrounded by a compromised host environment. As can be concluded from Table S1, the lack of muscular contribution to the vascularization of the fracture callus as well as of osteoprogenitor cells or growth factors, greatly hampers the bone regeneration process and results in the formation of a non-union. Furthermore, the initial conditions can be tuned to improve the amount of bone formation but are insufficient to provide complete healing of the critical size defect (Table S1). This was attributed to the delayed vascularization of the central callus area, leading to hypoxia and cell death. In order to improve the limited biological potential of the fracture callus and host environment, additional progenitor cells or growth factors can be injected in the fracture callus. However, cellular strategies would miss their therapeutic target if injections would take place at day 0, since cell survival would be very limited in these challenging hypoxic conditions. Therefore, we investigated whether a single injection of MSCs, osteochondrogenic growth factors or a combination thereof at a later time point would improve the bone healing outcome, as in this way the blood vessel network will have restored at least partially (Figure 13).

As can be seen in Figure 13, the injection of osteochondrogenic growth factors does not improve the bone healing outcome, except at PFD 0. This can be attributed to the increased chondrogenic differentiation and consequently limited oxygen consumption, as was discussed previously. At the other time points, the delay in vascularization of the central callus area results in hypoxia and cell death. Consequently, the injection of additional growth factors is to no avail since there are no cells present on which they can exert their influence. Interestingly, the time at which the MSCs or the combination product was injected, appears to be a critical determinant for the final amount of bone formation. If the cellular treatment is administered before PFD 35, the amount of bone is reduced (compared to no treatment) since the additional cells increase the oxygen consumption thereby worsening the hypoxic conditions in the central callus area. One can notice a further decrease of the effectiveness of the cellular treatment (cells only as well as the combination with growth factors) for injections at PFD 7 and 14. This can be related to the oxygen tension encountered in the central callus area at the time of injection, and the fact that this oxygen tension evolves with time. More specifically, at PFD 0 the oxygen tension has dropped only slightly and at PFD 28 the vasculature is already growing into the fracture callus so that in both cases the oxygen tension in the central callus area is able to support the injected cells. At intermediate time points, however, the oxygen tension is too low to support the injected cells, explaining why injections at PFD 7 and 14 are the least effective. If cells are administered at PFD 35, the delay of 35 days between the occurrence of the fracture and the start of the cellular therapy allows for a partial restoration of the blood vessel network, which seems to be optimal for the injection of cells only. The effectiveness of the combination product, however, continues to increase when the treatment is further postponed (up to day 56). The non-linearities in the predicted bone tissue fractions as a function of time of administration, as well as the discrepancy in optimal timing between the cellular and combination treatment can again be explained by the evolving oxygen tension of the central callus area which gradually increases as a function of time through a combination of oxygen release from the active vasculature and passive diffusion. More specifically, the average oxygen tension in the central callus area of a large non-treated defect surrounded by a compromised environment increases from 2.2% at 35 days, to 3% at 42 days, 3.9% at 49 days, 5.6% at 56 days and 6.2% at 63 days. At PFD 35 both the oxygen tension as well as the osteochondrogenic growth factor concentration are low in the central callus area so that only limited chondrogenic differentiation occurs upon injection of MSCs. However, the low oxygen tension inhibits extensive cellular proliferation (avoiding too much oxygen consumption), resulting in a small amount of “quiescent” stem cells (similar to *n_init_* = 0.7%, Table S1). When the oxygen tension in the central callus area subsequently increases to 3%, the remaining MSCs differentiate to chondrocytes and contribute to the bone regeneration process. As such, there are two bursts of chondrogenic differentiation which results in an increased amount of bone formation. If the cellular administration occurs after PFD 35 the increased oxygen tension will enhance the chondrogenic differentiation, thereby reducing or even eliminating the pool of “quiescent” stem cells. As such the proliferation and survival of the newly formed chondrocytes will mainly determine the extent of the bone formation. Since the average oxygen tension in the central callus area is higher at PFD 56 and 63 than at PFD 49, more bone will be formed for cellular injections in the former two cases, compared to PFD 49.

By combining osteochondrogenic growth factors with stem cells, all the injected MSCs will directly become chondrocytes thereby depleting the pool of osteoprogenitor cells, irrespective of the starting time of the treatment. Similar to the cellular treatments started after PFD 35 the proliferation and survival of the chondrocytes will mainly determine the extent of the bone formation. It appears that the average oxygen tension at PFD 56 results in an optimal proliferation and survival of the chondrocytes and hence subsequent endochondral bone formation. At PFD 63 the average oxygen tension becomes too high for optimal chondrocytic proliferation thereby reducing the amount of bone formation.

According to the model results, cellular injections are only effective if delayed until a specific time point (i.e. day 35, Figure 13) in order to allow for a partial restoration of the blood vessel network. Note that although the cellular as well as the combination treatment lead to an increased amount of bone compared to no treatment (provided the injection is sufficiently delayed), they nevertheless result in the formation of a non-union (with a maximal amount of bone at 90 days up to 80% and 85% respectively). As such, a single injection is insufficient and future research should focus on an optimal sequence of injections in order to heal critical sized defects in a compromised environments.

## Discussion

This study has used an integrative *in vivo* - *in silico* approach to investigate the occurrence of oligotrophic and atrophic non-unions as well as to design possible treatment strategies thereof. An extensive sensitivity analysis was performed in order to study the complex interplay of blood vessel formation, oxygen supply, growth factors and (osteoprogenitor) cells on the final healing outcome in large bone defects. The results of the sensitivity analysis indicated that the initial conditions (osteochondrogenic growth factor, MSCs, oxygen) are necessary for the bone regeneration process but not sufficient for complete bone healing of a critical size defect (5 mm). They do, however, have an important impact on the final amount of bone formation. Interestingly, simulation results of the same oxygen model in a small defect (0.5 mm) were found to be robust to changes in the initial conditions [23].

Although the performed sensitivity analysis yields interesting results, the interpretation thereof should be done carefully due to a number of reasons. For example, the sensitivity analysis that was performed in this study (Table S1) used a one-at-a-time (OAT) design, where the effect of one factor is assessed by varying the value of only that factor and keeping all other factors fixed. The main disadvantage of this simple method is its inability to capture interactions between factors. A simple combination of the ‘optimal’ values of initial conditions (see Table S1: the value that for an OAT design yielded the most bone at PFD 90) indicates for instance that a more adequate design is necessary to unravel these (non-linear) interactions between the different parameters of the oxygen model. Indeed, combining the ‘optimal’ initial conditions of MSCs (*c_m,init_*), fibroblasts (*c_f,init_*), osteochondrogenic growth factors (*g_bc,init_*) and oxygen (*n_init_*) results in 38% of bone after 90 days which is less than for the respective ‘optimal’ initial conditions alone. The conclusions of the sensitivity analysis are also only valid for this specific set of parameter values since, for example, the optimal initial oxygen tension will vary depending on the initial stem cell concentration. Despite its limitations, the OAT-design already indicates some interesting non-linear responses of the model with respect to the initial MSC cell density and the oxygen tension as well as their interactions. Future work should focus on more complex designs, including latin hypercube design and uniform design [41], to calculate quantitative metrics of sensitivity and study these non-linearities and (higher-order) parameter interactions further in order to unravel the underlying mechanisms and define new research hypotheses.

The dynamics of all cellular variables (apart from the endothelial cells) is described by means of continuum equations, meaning amongst others that cell proliferation was captured by means of a logistic growth equation. While this equation accounts for a maximal cell density in the callus area, it does not allow to specify an upper limit to the number of division cycles a cell (such as an MSC) can undergo before senescence. Because of this upper limit, in reality the amount of cells that can be obtained through division is dependent on the original pool size whereas in the mathematical model, a single cell can theoretically divide until the entire callus reaches maximal cell density. The main consequence of this limitation is that our predictions might be too optimistic in that fracture healing might be even more challenging in reality, because a sufficient number of cells (such as MSCs) cannot be reached to heal the fracture. In the future we will try to implement a description that allows to account for a limited number of population doublings, potentially through the extension of the agent-based description of endothelial cells to the skeletal cell types.

Even though the predictions of the current model might be too optimistic, all of the conditions explored in Table S1 nevertheless resulted in the formation of a non-union. Indeed, the simulation predicts that a murine bone defect becomes critical at 3 mm (Figure 6) which corresponds to the experimental observation of Zwingenberger et al. [42]. They report the creation of a persisting femoral bone defect in nude mice when the defect size is 3 mm [42]. The predicted value is also in the same range as other mouse femoral critical defect sizes reported in the literature: 2 mm [43], 3.5 mm [44] and 4 mm [45]. As such, the computational framework is able to model the occurrence of non-unions and can be used to design several treatment strategies depending on the host environment. In our model, a single initial (i.e. at PFD 0) injection of osteochondrogenic growth factors at sufficiently high concentration (*g_bc,init_* = 1 µg/ml) directly into a callus surrounded by a permissive environment resulted in complete healing of the critical size defect (Figure 9). The beneficial effect of growth factor delivery was also confirmed by the study of Patel et al. [46]. They report that the BMP-2 release from gelatin microparticles incorporated within the pores of a scaffold that was implanted within a 8 mm rat cranial critical defect resulted in significantly higher bone formation after 12 weeks, i.e. 37.4±18.8% (test) versus 7.8±7.1% (control) bone volume respectively. Similar conclusions were made by Willett et al. who studied the influence of recombinant human BMP-2 (rhBMP-2) delivery on tissue regeneration in a murine composite injury model [47]. The *in vivo* composite injury model consisted of a critically sized femoral bone defect and an adjacent volumetric muscle injury in the quadriceps (both 8 mm) [47]. They have shown that treated bone defects without volumetric muscle loss were consistently bridged whereas the treatment failed to promote the regeneration process in the challenging composite injury [47]. Although care must be taken when directly comparing these findings to our *in silico* results (since the exact role of the muscle in the *in vivo* setting of Willett et al. was not characterized), they do predict the same trends. Indeed, the multiscale model predicts a successful healing in the case of growth factor administration to a critical sized defect that is fully or partially supplied by blood vessels from the overlying muscle (Figure 9). In contrast, in a compromised environment where the role of the muscle as a source of vascularization is lacking, additional injections of growth factors, either at PFD 0 (Table S1) or at later time points (Figure 13) do not induce bony bridging of the large bone defect.

In large bone defects not only the initial concentration of growth factors but also the initial amount of osteoprogenitor cells might be reduced [26], [27]. Consequently, the use of stem cells for the treatment of critical size defects is actively being pursued [48]. The injection of MSCs in the callus area elicited an improved healing response (although without reaching full bridging) *in silico* if the environment is sufficiently vascularized to sustain the cell viability, which according to the model meant that injections were only effective if delayed until a certain time point (day 35 according to Figure 13). Similar conclusions were drawn by Geris et al. who investigated the occurrence of bone atrophic non-unions by an integrative approach [49]. Based on the recovery of the blood supply to the interfragmentary gap, they predicted with an *in silico* model that the injection of MSCs at three weeks post-osteotomy would prevent the onset of an atrophic non-union which was also confirmed by experimental results [49]. The necessity of vascularization for successful healing of challenging critical size defects is also substantiated by the results of Table S1 (no contribution of the overlying muscle to the vasculature) and Figure 9 (partial contribution of the overlying muscle to the vasculature), where an initial injection (i.e. at PFD 0) of additional cells in a defect that is insufficiently vascularized does not significantly improve the bone formation outcome. As such, the mathematical model retrieves the beneficial effect of cellular injections in some cases, similar to the experimental observations reported in literature [50], [51], although the effectiveness is strongly dependent on the available vasculature.

Interestingly, the model results indicate that the effectiveness of a therapy (consisting of the injection of cells, growth factors or a combination thereof) is dependent on the timing of the treatment as well as the host environment. The former effect is strongly related to the biological potential of the fracture callus at the time the treatment is applied, while the latter potentially constitutes a source of additional osteoprogenitor cells, growth factors or vascularization. For example, growth factor injections at PFD 0 or at later time points in a compromised host environment lead to only 63% and 52% of bone respectively whereas growth factor injections at PFD 0 in a permissive environment result in the formation of a union. In all three cases the main cause underlying the formation of a non-union in a large defect (without treatment) is the increased cell death in the central (hypoxic) callus area. Since growth factor injections at PFD 0 result in increased chondrogenic differentiation, which in turn limits the oxygen consumption and the decrease of oxygen tension (severity of hypoxia), this treatment increases the amount of bone formation. Note that nevertheless a hypoxic area arises which results in the formation of a non-union. Consequently, a permissive environment that provides additional vascular ingrowth, improves the bone formation outcome even further. Growth factor injections at later time points in a compromised environment are, however, to no avail since there are no cells left in the central callus area.

**Figure 14:**
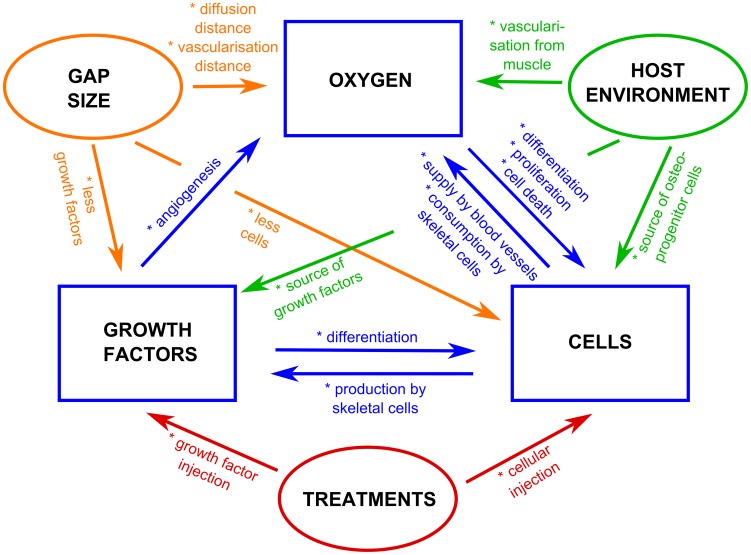
Schematic overview of the complex spatiotemporal interplay between the amount of oxygen, growth factors and cells as well as the gap size, the host environment and the administered treatments.

In summary, we can state that a treatment will be most beneficial if it tackles the underlying mechanism of action causing the hampered bone formation. Although this statement seems a logical and intuitive design rule, the underlying mechanisms of actions are a result of the complex non-linear, oxygen-dependent dynamics of blood vessel formation, oxygen supply, angiogenic growth factor production, cell differentiation, cell proliferation and oxygen consumption. The fact that many cellular processes, like survival, proliferation and differentiation are (non-linearly) dependent on oxygen tension and that they all have a specific range of oxygen tension at which they are ‘optimized’ (maximally affected) (Figures 1–2), makes it virtually impossible to intuitively predict the resulting bone healing outcomes. Instead, it requires a rigorous computational modelling of the governing mechanisms and dependencies (Figure 14).

Taken all the results together, we can conclude that complete cortical bridging of a challenging critical size defect will only occur if growth factors, osteoprogenitor cells and vasculature are present at the same time and place (Figure 14). Indeed, the blood vessels will supply the necessary oxygen to ensure cellular survival whereas the growth factors will promote the correct differentiation cascade finally resulting in the continuation and successful completion of the bone regeneration process. Consequently, the most stringent factor that is lacking in a certain area or at a certain time point will be an ideal candidate for potential treatment strategies. For example, bone tissue engineering treatments where a scaffold seeded with cells and osteochondrogenic growth factors is implanted in a bone defect, should focus on a timely vascularization in order to ensure the survival of the implanted cells. Potential strategies of vascularization include the induction of a Masquelet-membrane [52], [53], the delivery of angiogenic growth factors [46] as well as the *in vitro* creation of a pre-vascularized construct by co-culture of osteoprogenitor cells with endothelial cells [54]. Encouraging results were for example obtained by Patel et al. who showed that the dual release of vascular endothelial growth factor (VEGF) and bone morphogenetic protein-2 (BMP-2) in a 8 mm rat cranial critical size defect enhanced the bone formation at 4 weeks, suggesting a synergistic effect of these growth factors during early bone regeneration [46]. Note that besides the biological stimuli also mechanoregulatory stimuli influence the bone formation process [36], [37]. The current multiscale model does not take this into account, meaning amongst others one assumes that the fracture is sufficiently stabilized through external or internal fixation such that excessive loading will not play a role in the formation of a non-union (Supporting Text S2).

In conclusion, the multiscale oxygen model was able to capture the essential aspects of *in vivo* atrophic and oligotrophic non-unions. Interestingly, thorough model analyses assisted in understanding the underlying mechanisms of action, i.e. the delayed vascularization of the central callus region resulted in harsh hypoxic conditions, cell death and finally disrupted bone healing. Since a timely vascularization was found to be critical for the successful healing of large bone defects, the oxygen model was used to design and test potential treatment strategies for both permissive and compromised host environments. A qualitative correspondence between the predicted outcomes of certain treatment strategies and experimental observations was obtained, clearly illustrating the model's potential. Furthermore, the results of this study demonstrate that due to the complex non-linear, oxygen-dependent dynamics of blood vessel formation, oxygen supply, angiogenic growth factor production, cell differentiation, cell proliferation and oxygen consumption, it becomes virtually impossible to determine the effectiveness of a treatment strategy intuitively thereby underlining the importance computational modelling tools. Moreover, the model predictions also showed that the effectiveness of a therapy is strongly influenced by the host environment since it can serve as a source of additional osteoprogenitor cells, growth factors or vascularization to populate the fracture callus and increase the biological potential thereof. Consequently, future research should focus on extensive experimental characterization as well as computational modelling of the host environment and its interaction with potential treatment strategies.

## Supporting Information

Figure S1 **Graphical representation of the initial position of the ECs for six different simulation cases.**
(TIF)

Figure S2 **The predicted spatiotemporal evolution of fracture healing in a critical sized defect (5 mm) for different values of the diffusion coefficient of oxygen.** (A–D–G) bone matrix density (×0.1 g/ml), (B–E–H) oxygen tension (×1%) and (C–F–I) active vasculature for different values of the diffusion coefficient of oxygen (D_n_): (A–B–C) 2.10^−13^ m^2^/s, (D–E–F) 2.10^−11^ m^2^/s, (G–H–I) 2.10^−10^ m^2^/s (Table S1).
(TIF)

Figure S3 **The predicted spatiotemporal evolution of fracture healing in different host environments.** (i) bone matrix density (×0.1 g/ml), (ii) active vasculature. (case A) the overlying muscle fully contributes to the ingrowing vasculature, (case B) the overlying muscle partially contributes to the ingrowing vasculature, (case C) the overlying muscle produces growth factors over the entire length of the gap and (case D) the overlying muscle delivers osteoprogenitor cells over the entire length of the gap (Figure 8).
(TIF)

Table S1 **Overview of the results of the sensitivity analysis on the initial conditions and the parameter values describing oxygen delivery, oxygen diffusion and oxygen consumption.**
(DOCX)

Table S2 **Influence of the muscle as a source for vascularization, MSCs, growth factors or a combination thereof on the bone regeneration process.**
(DOCX)

Table S3 **Results of three types of treatment strategies in a permissive host environment where the overlying muscle partially contributes to the vasculature of the fracture callus.**
(DOCX)

Text S1 **Description of the mathematical model.**
(DOCX)

Text S2 **Estimation of the interfragmentary strains.**
(DOCX)

Text S3 **Influence of the diffusion coefficient of oxygen.**
(DOCX)

Text S4 **Influence of the host environment.**
(DOCX)
