# Supplementary figures and images for: Size Does Matter: An Integrative In Vivo-In Silico Approach for the Treatment of Critical Size Bone Defects
Source: PLoS Comput Biol. 2014 Nov 6;10(11):e1003888. doi: 10.1371/journal.pcbi.1003888 (PMC4222588; doi:10.1371/journal.pcbi.1003888)

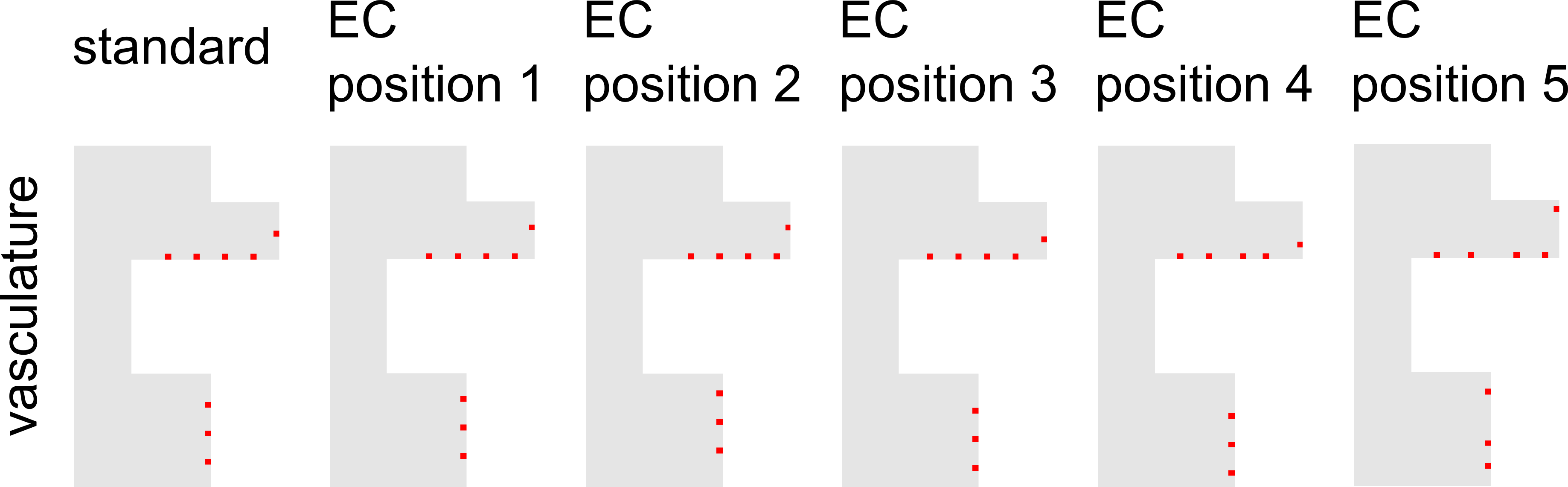

Supplement: Figure S1 — Graphical representation of the initial position of the ECs for six different simulation cases. (TIF) [file pcbi.1003888.s001.tif]

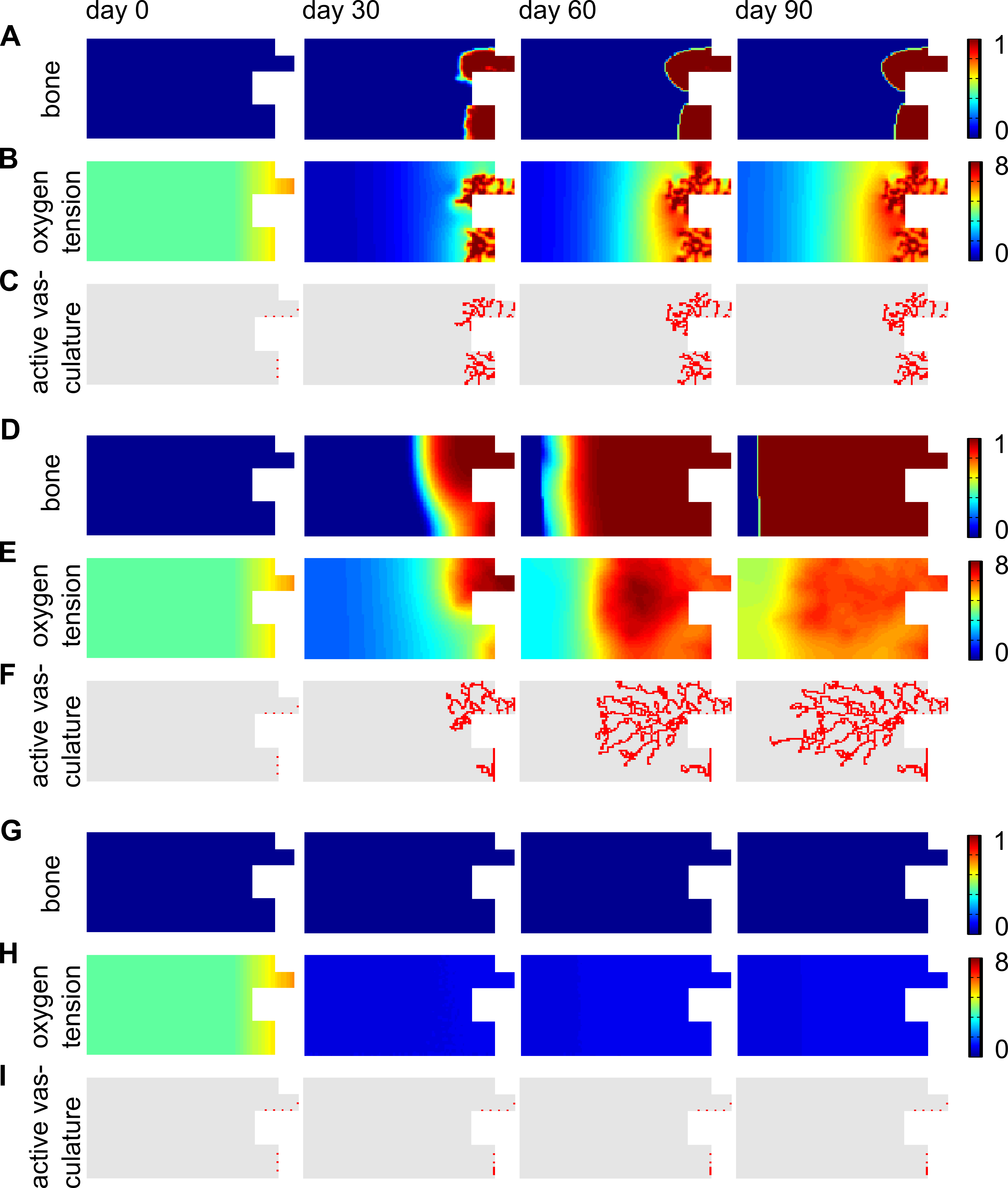

Supplement: Figure S2 — The predicted spatiotemporal evolution of fracture healing in a critical sized defect (5 mm) for different values of the diffusion coefficient of oxygen. (A–D–G) bone matrix density (×0.1 g/ml), (B–E–H) oxygen tension (×1%) and (C–F–I) active vasculature for different values of the diffusion coefficient of oxygen (Dn): (A–B–C) 2.10−13 m2/s, (D–E–F) 2.10−11 m2/s, (G–H–I) 2.10−10 m2/s (Table S1). (TIF) [file pcbi.1003888.s002.tif]

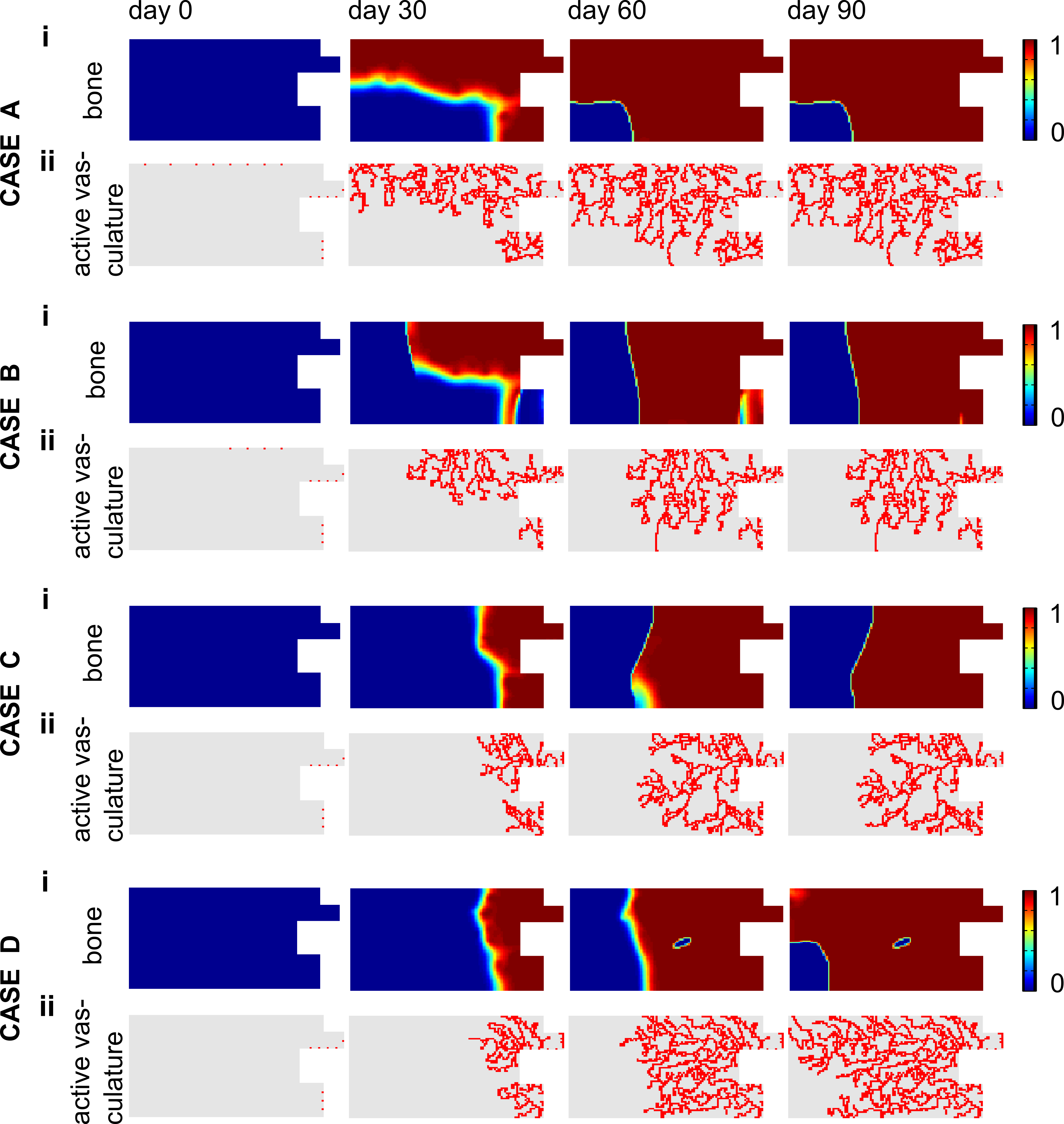

Supplement: Figure S3 — The predicted spatiotemporal evolution of fracture healing in different host environments. (i) bone matrix density (×0.1 g/ml), (ii) active vasculature. (case A) the overlying muscle fully contributes to the ingrowing vasculature, (case B) the overlying muscle partially contributes to the ingrowing vasculature, (case C) the overlying muscle produces growth factors over the entire length of the gap and (case D) the overlying muscle delivers osteoprogenitor cells over the entire length of the gap (Figure 8). (TIF) [file pcbi.1003888.s003.tif]
