# Supplementary material for: Size Does Matter: An Integrative In Vivo-In Silico Approach for the Treatment of Critical Size Bone Defects
Source: PLoS Comput Biol. 2014 Nov 6;10(11):e1003888. doi: 10.1371/journal.pcbi.1003888 (PMC4222588; doi:10.1371/journal.pcbi.1003888)
Supplement: Table S1 — Overview of the results of the sensitivity analysis on the initial conditions and the parameter values describing oxygen delivery, oxygen diffusion and oxygen consumption. (DOCX) [file pcbi.1003888.s004.docx]

**Table S1: Overview of the results of the sensitivity analysis on the initial conditions and the parameter values describing oxygen delivery, oxygen diffusion and oxygen consumption.** The tissue fractions are measured at post fracture day (PFD) 90. The dimensionalized parameter values corresponding to the uniform, standard initial conditions in the central area of the callus (indicated with dots in Figure 3) are *c_m,init_* = 2.10^3^ cells/ml, *g_bc,init_* = 10 ng/ml, *c_f,init_* = 1.10^4^ cells/ml, *m_f,init_* =0.01 g/ml*, n_init_* = 3.7%. The parameter values related to oxygen delivery, diffusion and consumption are *G_n_* = 2.2.10^-12^ mol/cell.day (oxygen production rate), *D_n_* = 2.10^-12^ m^2^/s (oxygen diffusion coefficient), *Q_b_* = 25.5.10^-18^ mol/cell.s (maximal oxygen consumption rate of osteoblasts), *Q_c_* = 0.464.10^-18^ mol/cell.s (maximal oxygen consumption rate of chondrocytes), *Q_m_* = 23.2.10^-18^ mol/cell.s (maximal oxygen consumption rate of MSCs), *Q_f_* = 29.10^-18^ mol/cell.s (maximal oxygen consumption rate of fibroblasts). The complete set of non-dimensionalized parameter values can be found in the supplementary material. *n_init,gr_* = 0.8%/mm*x with x the coordinate on the horizontal axis in Figure 3 (in mm). The standard condition is indicated in bold.

| **Condition** | | **Bone** | **Fibrous matrix** | **Cartilage matrix** |
| --- | --- | --- | --- | --- |
| **standard compromised condition** | | **52%** | **48%** | **0%** |
| EC position 1 | | 51% | 49% | 0% |
| EC position 2 | | 50% | 50% | 0% |
| EC position 3 | | 51% | 49% | 0% |
| EC position 4 | | 52% | 48% | 0% |
| EC position 5 | | 48% | 52% | 0% |
| *c_m,init_* | 1.10^2^ cells/ml | 39% | 61% | 0% |
|  | 2. 10^2^ cells/ml | 44% | 56% | 0% |
|  | 2.10^4^ cells/ml | 49% | 51% | 0% |
|  | 2.10^5^ cells/ml | 51% | 49% | 0% |
|  | 1.10^6^ cells/ml | 42% | 58% | 0% |
| *g_bc,init_* | 1 ng/ml | 49% | 51% | 0% |
|  | 1.10^2^ ng/ml | 60% | 40% | 0% |
|  | 1.10^3^ ng/ml | 63% | 37% | 0% |
| *c_f,init_* | 1. 10^3^ cells/ml | 50% | 50% | 0% |
|  | 1. 10^5^ cells/ml | 55% | 45% | 0% |
|  | 5.10^5^ cells/ml | 39% | 59% | 2% |
|  | 1.10^6^ cells/ml | 30% | 70% | 0% |
| *m_f,init_* | 1.10^-3^ g/ml | 47% | 53% | 0% |
|  | 1.10^-1^ g/ml | 37% | 63% | 0% |
| *n_init_* | 0.1% | 35% | 65% | 0% |
|  | 0.3% | 42% | 58% | 0% |
|  | 0.7% | 74% | 26% | 0% |
|  | 1.2% | 61% | 39% | 0% |
|  | 1.7% | 56% | 44% | 0% |
|  | 2.7% | 56% | 44% | 0% |
|  | 4.7% | 48% | 52% | 0% |
|  | 5.7% | 45% | 55% | 0% |
| *n_init,gr_* | 0.8%/mm*x | 65% | 35% | 0% |
| *c_m,init_ - g_bc,init_*  *$\tilde{g}_{\mathrm{bc},init}$* | 2.10^4^ cells/ml - 1.10^3^ ng/ml | 47% | 53% | 0% |
| *$\tilde{g}_{\mathrm{bc},init}$* | 2.10^5^ cells/ml - 1.10^3^ ng/ml | 40% | 60% | 0% |
| *c_m,init_ - c_f,init_ - g_bc,init_ - n_init_* | 2.10^5^ cells/ml - 1.10^5^ cells/ml  - 1.10^3^ ng/ml - 0.7% | 38% | 62% | 0% |
| *G_n_* | 0.22.10^-12^ mol/cell.day | 0% | 88% | 12% |
|  | 1.2.10^-12^ mol/cell.day | 49% | 51% | 0% |
|  | 3.2.10^-12^ mol/cell.day | 55% | 45% | 0% |
|  | 22 .10^-12^ mol/cell.day | 37% | 63% | 0% |
| *D_n_* | 2.10^-13^ m^2^/s | 14% | 86% | 0% |
|  | 2.10^-11^ m^2^/s | 89% | 11% | 0% |
|  | 2.10^-10^ m^2^/s | 0% | 100% | 0% |
| *Q_b_* | 2.55.10^-18^ mol/cell.s | 52% | 48% | 0% |
|  | 13.92.10^-18^ mol/cell.s | 52% | 48% | 0% |
|  | 37.12.10^-18^ mol/cell.s | 51% | 49% | 0% |
|  | 255.2.10^-18^ mol/cell.s | 16% | 54% | 30% |
| *Q_c_* | 0.0464.10^-18^ mol/cell.s | 62% | 38% | 0% |
|  | 0.348.10^-18^ mol/cell.s | 49% | 51% | 0% |
|  | 0.58.10^-18^ mol/cell.s | 46% | 54% | 0% |
|  | 4.64.10^-18^ mol/cell.s | 5% | 90% | 5% |
| *Q_m_* | 2.32.10^-18^ mol/cell.s | 60% | 40% | 0% |
|  | 11.6 .10^-18^ mol/cell.s | 57% | 43% | 0% |
|  | 34.8.10^-18^ mol/cell.s | 48% | 52% | 0% |
|  | 232.10^-18^ mol/cell.s | 15% | 85% | 0% |
| *Q_f_* | 2.9.10^-18^ mol/cell.s | 52% | 48% | 0% |
|  | 17.4.10^-18^ mol/cell.s | 52% | 48% | 0% |
|  | 40.6.10^-18^ mol/cell.s | 51% | 49% | 0% |
|  | 290.10^-18^ mol/cell.s | 44% | 56% | 0% |
