# Supplementary material for: Size Does Matter: An Integrative In Vivo-In Silico Approach for the Treatment of Critical Size Bone Defects
Source: PLoS Comput Biol. 2014 Nov 6;10(11):e1003888. doi: 10.1371/journal.pcbi.1003888 (PMC4222588; doi:10.1371/journal.pcbi.1003888)
Supplement: Table S2 — Influence of the muscle as a source for vascularization, MSCs, growth factors or a combination thereof on the bone regeneration process. (DOCX) [file pcbi.1003888.s005.docx]

**Table S2: Influence of the muscle as a source for vascularization, MSCs, growth factors or a combination thereof on the bone regeneration process.** The tissue fractions are measured at post fracture day (PFD) 90. The standard condition is indicated in bold and has the following dimensionalized parameter values for the initial conditions in the central area of the fracture callus: *c_m,init_* = 2.10^3^ cells/ml, *g_bc,init_* = 10 ng/ml*, c_f,init_* = 1.10^4^ cells/ml, *m_f,init_* =0.01 g/ml, *n_init_* = 3.7%.

| **Condition** | | | | | **Bone** | **Fibrous matrix** | **Cartilage matrix** | **Union** |
| --- | --- | --- | --- | --- | --- | --- | --- | --- |
| **standard compromised condition** | | | | | **52%** | **48%** | **0%** |  |
|  | **Full vascularization** | **Partial vascularization** | **Growth factors** | **MSCs** |  |  |  |  |
| **A** | X |  |  |  | 88% | 12% | 0% | X |
| **B** |  | X |  |  | 66% | 34% | 0% |  |
| **C** |  |  | X |  | 59% | 41% | 0% |  |
| **D** |  |  |  | X | 90% | 10% | 0% | X |
| **E** |  |  | X | X | 71% | 28% | 1% | X |
| **F** | X |  | X |  | 100% | 0% | 0% | X |
| **G** | X |  |  | X | 84% | 16% | 0% | X |
| **H** | X |  | X | X | 100% | 0% | 0% | X |
| **I** |  | X | X |  | 91% | 9% | 0% | X |
| **J** |  | X |  | X | 85% | 15% | 0% | X |
| **K** |  | X | X | X | 100% | 0% | 0% | X |
