# Supplementary material for: Size Does Matter: An Integrative In Vivo-In Silico Approach for the Treatment of Critical Size Bone Defects
Source: PLoS Comput Biol. 2014 Nov 6;10(11):e1003888. doi: 10.1371/journal.pcbi.1003888 (PMC4222588; doi:10.1371/journal.pcbi.1003888)
Supplement: Table S3 — Results of three types of treatment strategies in a permissive host environment where the overlying muscle partially contributes to the vasculature of the fracture callus. (DOCX) [file pcbi.1003888.s006.docx]

**Table S3: Results of three types of treatment strategies in a permissive host environment where the overlying muscle partially contributes to the vasculature of the fracture callus.** The tissue fractions are measured at PFD 90. The standard condition is indicated in bold and has the following dimensionalized parameter values for the initial conditions in the central area of the callus: *c_m,init_* = 2.10^3^ cells/ml, *g_bc,init_* = 10 ng/ml, *c_f,init_* =1.10^4^ cells/ml, *m_f,init_* =0.01 g/ml, *n_init_* = 3.7%.

| **Condition** | | **Bone** | **Fibrous matrix** | **Cartilage matrix** | **Union?** |
| --- | --- | --- | --- | --- | --- |
| **standard permissive condition environment** | | **66%** | **34%** | **0%** |  |
| *c_m,init_* | 2.10^4^ cells/ml | 65% | 35% | 0% |  |
| *c_m,init_* | 2.10^5^ cells/ml | 68% | 32% | 0% |  |
| *g_bc,init_* | 1.10^2^ ng/ml | 78% | 22% | 0% |  |
| *g_bc,init_* | 1.10^3^ ng/ml | 89% | 11% | 0% | X |
| *g_bc,init_ / c_m,init_* | 1.10^2^ ng/ml / 2.10^4^ cells/ml | 77% | 23% | 0% |  |
| *g_bc,init_ / c_m,init_* | 1.10^2^ ng/ml / 2.10^5^ cells/ml | 76% | 24% | 0% |  |
| *g_bc,init_ / c_m,init_* | 1.10^3^ ng/ml / 2.10^4^ cells/ml | 73% | 27% | 0% |  |
| *g_bc,init_ / c_m,init_* | 1.10^3^ ng/ml / 2.10^5^ cells/ml | 71% | 27% | 2% |  |
