# Supplementary material for: Size Does Matter: An Integrative In Vivo-In Silico Approach for the Treatment of Critical Size Bone Defects
Source: PLoS Comput Biol. 2014 Nov 6;10(11):e1003888. doi: 10.1371/journal.pcbi.1003888 (PMC4222588; doi:10.1371/journal.pcbi.1003888)
Supplement: Text S2 — Estimation of the interfragmentary strains. (DOCX) [file pcbi.1003888.s008.docx]

# Estimation of the interfragmentary strains

Although a numerical model (such as a finite element model) would be needed to calculate detailed distributions of mechanical cues within the fracture callus, one can estimate the interfragmentary strain as follows (Figure S.2.1). If the callus and external fixator are regarded as parallel springs loaded by an axial force, then the fraction of that force carried by the callus is given by

$\frac{F_{callus}}{F_{total}}=\frac{k_{callus}}{k_{callus}+k_{fixator}}$ (S.35)

with *k_callus_* and *k_fixator_* the axial stiffness of the callus and the fixator respectively. While the fixator stiffness in the study of Harrison et al. [27] was measured to be 46 N/mm, the callus stiffness can be estimated as:

$k_{callus}=\frac{E}{Ad}$ (S.36)

with *E* the Young’s modulus of the callus tissue, *A* the callus cross-sectional area and *d* the gap size (either 0.5 or 3 mm). Taking the callus modulus equal to 0.2 MPa (as has been reported for granulation tissue [38]) this yields a (initial) callus stiffness of 7.9 and 1.3 N/mm for a gap size of 0.5 and 3 mm respectively (and assuming a callus diameter of 5 mm). The interfragmentary strain (IFS) can be calculated as follows

$IFS=\frac{u_{calllus}}{d}=\frac{F_{callus}}{k_{callus}d}$ (S.37)

For an axial femoral force of 10N (equaling twice the body weight of twelve-month-old male Wistar rats [27]) one can therefore estimate the interfragmentary strain to be 37 and 7% in case of the 0.5 and 3 mm gap size respectively. These numbers indeed suggest that for this animal model excessive loading did not play a role in the non-union of the critically sized defect. For other animals models it could nevertheless be interesting to explore the combination of mechanical and biological cues in one integrated model, similar to Geris et al. [39].


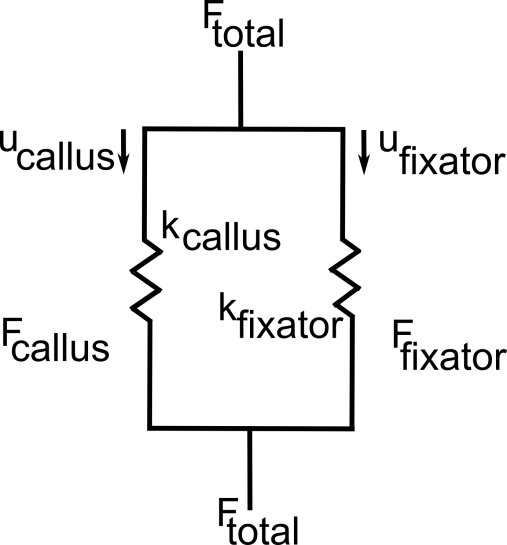


**Figure S.2.1: Schematical representation of the callus-fixator system.**
