# Supplementary material for: Size Does Matter: An Integrative In Vivo-In Silico Approach for the Treatment of Critical Size Bone Defects
Source: PLoS Comput Biol. 2014 Nov 6;10(11):e1003888. doi: 10.1371/journal.pcbi.1003888 (PMC4222588; doi:10.1371/journal.pcbi.1003888)
Supplement: Text S3 — Influence of the diffusion coefficient of oxygen. (DOCX) [file pcbi.1003888.s009.docx]

# Influence of the diffusion coefficient of oxygen

It can be noticed from Table S1 and Figure S2 that the diffusion properties of oxygen have a major impact on the simulation outcome. Indeed, reducing the diffusion coefficient of oxygen impairs the bone formation due to the creation of a larger hypoxic zone (Figure S2-A,C) whereas increasing the diffusion coefficient results in an almost uniform distribution of very low oxygen tensions across the entire callus, leading to cell death and impaired bone regeneration (Figure S2-H). As such, adequate oxygen diffusion is required to give rise to gradients in oxygen tension, thereby creating optimal environments of osteogenic and chondrogenic differentiation (although only in a restricted part of the callus).
