# Supplementary material for: Size Does Matter: An Integrative In Vivo-In Silico Approach for the Treatment of Critical Size Bone Defects
Source: PLoS Comput Biol. 2014 Nov 6;10(11):e1003888. doi: 10.1371/journal.pcbi.1003888 (PMC4222588; doi:10.1371/journal.pcbi.1003888)
Supplement: Text S4 — Influence of the host environment. (DOCX) [file pcbi.1003888.s010.docx]

# Influence of the host environment

Both the contribution of the muscle as a source of vascularization (case A) as well as of osteoprogenitor cells (case D) results in the formation of a union (Figure S3). In the first, the additional ingrowth of blood vessels reduces the hypoxic conditions thereby increasing the survival of the cells in the central callus area. In the latter, there is a continuous supply of new cells to ensure the continuation of the fracture healing process. Note that in both cases a small hole remains uncalcified in the left-bottom part of the central callus (Figure S3). Interestingly, the absence of bone formation in this left-bottom corner is not caused by a lack of oxygen or cells (since the overlying muscle is an additional source thereof) but by a lack of growth factors. The pool of growth factors that is initially present in the callus gradually diminishes through natural degradation and if not replenished by new growth factors produced by the chondrocytes and osteoblasts, the concentrations get too low to promote differentiation. As such, in the left-bottom corner the remaining MSCs do not commit to the chondrogenic lineage. The presence of this small hole is, however, of no importance since bridging of three of the four cortices clinically constitutes a union.

When the fracture callus is only partially supplied with blood vessels from the host environment (case B) or when the overlying muscle produces osteochondrogenic growth factors (case C), an increase in the amount of bone formation with respect to the standard compromised condition is seen but a complete bridging of the defect is not obtained (Figure S3). In these two cases, the insufficient supply of oxygen results in hypoxic conditions and cell death in the central fracture area.

In most cases, except for cases E and G, the combination of two or more boundary conditions enhances the bone formation process. Indeed, by combining for example the positive effects of additional oxygen delivery through the new vessels growing from the muscle with growth factor delivery, also the left-bottom corner will be calcified resulting in a completely bridged defect (case F). Likewise, the availability of oxygen, growth factors and precursor cells results in a successful completion of the bone regeneration process (case H). Interestingly, the combined delivery of cells and growth factors results in less bone formation (case E) than the delivery of cells alone (case D). This difference is due to the fact that the osteoprogenitor cells will differentiate immediately to chondrocytes or osteoblasts depending on the available oxygen tension. The depletion of the MSC pool, which (also in the mathematical model) has a migratory phenotype (compared to chondrocytes and osteoblasts who cannot migrate in the mathematical model), hampers the repopulation of the entire callus and finally the bone formation process.

The model simulations also predict that without vascular ingrowth from the muscular environment, the delivery of cells results in the largest amount of bone formation (case C versus case D). However, if the fracture callus is fully or partially vascularized by the overlying muscle, the delivery of growth factors is more beneficial than the delivery of cells for the final healing outcome (case F versus G, case I versus J). This non-intuitive result can be explained as follows. Without vascular ingrowth from the muscular environment, the bone formation process is mainly hampered by the increased cell death in the central callus area. As such, a continuous delivery of cells by the muscular environment greatly improves the fracture healing outcome (case D). Note, however, that in case D the MSCs in the left-bottom corner of the callus domain do not commit to the chondrogenic lineage due to the low concentrations of osteochondrogenic growth factor, resulting in a small uncalcified area as explained previously (Figure S3). While vascular ingrowth from the muscular environment leads to increased cell survival in the central callus area, the bone formation is mainly hampered by the low concentrations of osteochondrogenic growth factor in the left-bottom corner of the domain. Consequently, the combination of vascular ingrowth from the muscle with the delivery of osteochondrogenic growth factors will improve the bone healing outcome as it will also stimulate the formation of bone in the left-bottom corner of the domain (Figure S3).

The comparison of cases A, D and G yields another non-intuitive result. Apparently the combination of vascular ingrowth from the muscle with cell delivery (case G) results in less bone formation than the delivery of cells (case D) or vascular ingrowth (case A) alone. This difference is, similar to the combined delivery of growth factors and cells (case E), due to the localized differentiation of MSCs directly below the muscular envelope. As MSCs are the only migratory cells (together with fibroblasts) the repopulation of the fracture callus is hampered as well as the bone formation process.
